# Association of *FADS1/2* Locus Variants and Polyunsaturated Fatty Acids With Aortic Stenosis

**DOI:** 10.1001/jamacardio.2020.0246

**Published:** 2020-03-18

**Authors:** Hao Yu Chen, Benjamin J. Cairns, Aeron M. Small, Hannah A. Burr, Athithan Ambikkumar, Andreas Martinsson, Sébastien Thériault, Hans Markus Munter, Brian Steffen, Richard Zhang, Rebecca T. Levinson, Christian M. Shaffer, Jian Rong, Emily Sonestedt, Line Dufresne, Johan Ljungberg, Ulf Näslund, Bengt Johansson, Dilrini K. Ranatunga, Rachel A. Whitmer, Matthew J. Budoff, Albert Nguyen, Ramachandran S. Vasan, Martin G. Larson, William S. Harris, Scott M. Damrauer, Ken D. Stark, S. Matthijs Boekholdt, Nicholas J. Wareham, Philippe Pibarot, Benoit J. Arsenault, Patrick Mathieu, Vilmundur Gudnason, Christopher J. O’Donnell, Jerome I. Rotter, Michael Y. Tsai, Wendy S. Post, Robert Clarke, Stefan Söderberg, Yohan Bossé, Quinn S. Wells, J. Gustav Smith, Daniel J. Rader, Mark Lathrop, James C. Engert, George Thanassoulis

**Affiliations:** 1 Division of Experimental Medicine, McGill University, Montreal, Quebec, Canada; 2 Preventive and Genomic Cardiology, McGill University Health Centre and Research Institute, Montreal, Quebec, Canada; 3 MRC (Medical Research Council) Population Health Research Unit, Nuffield Department of Population Health, University of Oxford, Oxford, United Kingdom; 4 Clinical Trial Service Unit, Nuffield Department of Population Health, University of Oxford, Oxford, United Kingdom; 5 Epidemiological Studies Unit, Nuffield Department of Population Health, University of Oxford, Oxford, United Kingdom; 6 Department of Medicine, Perelman School of Medicine, University of Pennsylvania, Philadelphia; 7 Department of Cardiology, Clinical Sciences, Lund University, Lund, Sweden; 8 Department of Cardiology, Skåne University Hospital, Lund, Sweden; 9 Quebec Heart and Lung Institute, Laval University, Quebec City, Quebec, Canada; 10 McGill University and Genome Quebec Innovation Centre, Montreal, Quebec, Canada; 11 Department of Laboratory Medicine and Pathology, University of Minnesota, Minneapolis, Minnesota; 12 Vanderbilt Translational and Clinical Cardiovascular Research Center, Vanderbilt University Medical Center, Nashville, Tennessee; 13 National Heart, Lung, and Blood Institute, Bethesda, Maryland; 14 Boston University’s Framingham Heart Study, Boston, Massachusetts; 15 Nutritional Epidemiology, Department of Clinical Sciences Malmö, Lund University, Malmö, Sweden; 16 Department of Public Health and Clinical Medicine, Umeå University, Umeå, Sweden; 17 Division of Research, Kaiser Permanente of Northern California, Oakland; 18 Department of Public Health Sciences, University of California, Davis; 19 Los Angeles Biomedical Research Institute, Torrance, California; 20 Departments of Pediatrics and Medicine at Harbor-UCLA (University of California, Los Angeles) Medical Center, Torrance; 21 Department of Medicine, Sanford School of Medicine, University of South Dakota, Sioux Falls, South Dakota; 22 OmegaQuant Analytics LLC, Sioux Falls, South Dakota; 23 Department of Surgery, Perelman School of Medicine, University of Pennsylvania, Philadelphia; 24 Department of Kinesiology, University of Waterloo, Waterloo, Ontario, Canada; 25 Department of Cardiology, Amsterdam University Medical Center, University of Amsterdam, Amsterdam, the Netherlands; 26 MRC Epidemiology Unit, University of Cambridge, Cambridge, United Kingdom; 27 Faculty of Medicine, University of Iceland, Reykjavík; 28 Division of Cardiology, Department of Medicine, The Johns Hopkins University School of Medicine, Baltimore, Maryland; 29 Wallenberg Center for Molecular Medicine, Lund University, Lund, Sweden; 30 Lund University Diabetes Center, Lund University, Lund, Sweden; 31 Department of Genetics, Perelman School of Medicine, University of Pennsylvania, Philadelphia; 32 Department of Medicine, Perelman School of Medicine, University of Pennsylvania, Philadelphia; 33 Department of Human Genetics, McGill University, Montreal, Quebec, Canada

## Abstract

**Importance:**

Aortic stenosis (AS) has no approved medical treatment. Identifying etiological pathways for AS could identify pharmacological targets.

**Objective:**

To identify novel genetic loci and pathways associated with AS.

**Design, Setting, and Participants:**

This genome-wide association study used a case-control design to evaluate 44 703 participants (3469 cases of AS) of self-reported European ancestry from the Genetic Epidemiology Research on Adult Health and Aging (GERA) cohort (from January 1, 1996, to December 31, 2015). Replication was performed in 7 other cohorts totaling 256 926 participants (5926 cases of AS), with additional analyses performed in 6942 participants from the Cohorts for Heart and Aging Research in Genomic Epidemiology (CHARGE) Consortium. Follow-up biomarker analyses with aortic valve calcium (AVC) were also performed. Data were analyzed from May 1, 2017, to December 5, 2019.

**Exposures:**

Genetic variants (615 643 variants) and polyunsaturated fatty acids (ω-6 and ω-3) measured in blood samples.

**Main Outcomes and Measures:**

Aortic stenosis and aortic valve replacement defined by electronic health records, surgical records, or echocardiography and the presence of AVC measured by computed tomography.

**Results:**

The mean (SD) age of the 44 703 GERA participants was 69.7 (8.4) years, and 22 019 (49.3%) were men. The rs174547 variant at the *FADS1/2* locus was associated with AS (odds ratio [OR] per C allele, 0.88; 95% CI, 0.83-0.93; *P* = 3.0 × 10^−6^), with genome-wide significance after meta-analysis with 7 replication cohorts totaling 312 118 individuals (9395 cases of AS) (OR, 0.91; 95% CI, 0.88-0.94; *P* = 2.5 × 10^−8^). A consistent association with AVC was also observed (OR, 0.91; 95% CI, 0.83-0.99; *P* = .03). A higher ratio of arachidonic acid to linoleic acid was associated with AVC (OR per SD of the natural logarithm, 1.19; 95% CI, 1.09-1.30; *P* = 6.6 × 10^−5^). In mendelian randomization, increased *FADS1* liver expression and arachidonic acid were associated with AS (OR per unit of normalized expression, 1.31 [95% CI, 1.17-1.48; *P* = 7.4 × 10^−6^]; OR per 5–percentage point increase in arachidonic acid for AVC, 1.23 [95% CI, 1.01-1.49; *P* = .04]; OR per 5–percentage point increase in arachidonic acid for AS, 1.08 [95% CI, 1.04-1.13; *P* = 4.1 × 10^−4^]).

**Conclusions and Relevance:**

Variation at the *FADS1/2* locus was associated with AS and AVC. Findings from biomarker measurements and mendelian randomization appear to link ω-6 fatty acid biosynthesis to AS, which may represent a therapeutic target.

## Key Points

**Question:**

Can genetic analysis identify additional causes of aortic stenosis?

**Findings:**

In this genome-wide association study of 44 703 participants, each copy of a *FADS1/2* (fatty acid desaturase) genetic variant was associated with a 13% decrease in the odds of aortic stenosis. Results of a meta-analysis with 7 replication cohorts showed genome-wide significance, with biomarker and mendelian randomization analyses implicating elevated ω-6 fatty acid levels as having a potentially causal association with aortic valve calcium and aortic stenosis.

**Meaning:**

These findings demonstrate that the *FADS1/2* locus and fatty acid biosynthesis are associated with aortic stenosis and should be examined further for their potential as therapeutic targets.

## Teaser

This genome-wide association study identifies genetic loci and pathways associated with aortic stenosis.

## Introduction

Aortic stenosis (AS) remains the leading cause of clinical valve disease in the developed world.^1^ Contemporary treatment is limited to replacement of the aortic valve, because no approved medical therapy currently exists. The development of such therapy would expand options for treating AS but is hindered by a limited understanding of the causal contributors.

A genome-wide association study (GWAS) conducted in the Cohorts for Heart and Aging Research in Genomic Epidemiology (CHARGE) Consortium demonstrated that the *LPA* locus (OMIM 152200), which codes for the apolipoprotein(a) moiety of lipoprotein(a), is causally associated with incident AS and prevalent aortic valve calcium (AVC), a subclinical phenotype that precedes AS.^2^ This association with AS has been robustly confirmed in multiple cohorts.^3,4,5^ Recent clinical trials have demonstrated that significant reductions in lipoprotein(a) levels are achievable,^6,7,8^ which may represent a novel AS prevention strategy. Two recent GWAS (≤2457 cases of AS) have identified *TEX41* and *PALMD* variants as associated with AS, implicating abnormal cardiac development in disease etiology.^9,10^

A GWAS with a greater number of cases could have improved statistical power to discover additional genetic loci for AS and identify novel pathways as pharmacological targets. Accordingly, we performed a GWAS for AS among 44 703 participants (3469 cases of AS) from the Genetic Epidemiology Research on Adult Health and Aging (GERA) cohort, one of the largest collections of AS cases in the world. We validated our findings in 7 additional cohorts totaling 256 926 participants (5926 cases of AS) and performed genetic and plasma biomarker analyses to describe a novel mechanism underlying AS, with potential therapeutic implications. An overview of the study is given in eFigure 1 in the Supplement.

## Methods

### Genetic Discovery and Replication

In the GERA cohort (NCBI Database of Genotypes and Phenotypes, phs000788.v2.p3), we performed a GWAS for prevalent AS (615 643 variants), adjusting for age, age squared, and sex, among 44 703 unrelated individuals of self-reported European ancestry, 55 years or older (eTable 1 in the Supplement). We restricted our analysis to European participants owing to the small numbers of non-European GERA participants, because differences in genetic structure between ancestries may confound our findings. Aortic stenosis status was ascertained through electronic health records (January 1, 1996, to December 31, 2015, inclusive), using the *International Classification of Diseases, 9th Revision*, diagnosis code for AS (*ICD-9*) (424.1) or a procedure code for aortic valve replacement to designate cases; all other individuals were designated as controls. Individuals with congenital valvular disease (*ICD-9* codes 746-747) were excluded. Details of this case-control study are given in eMethods in the Supplement. All participants have provided written, informed consent, and all relevant internal review boards approved this study. This study followed the Strengthening the Reporting of Genetic Association Studies (STREGA) reporting guideline.

We later received updated GERA data for 55 192 unrelated participants of European-ancestry 55 years and older (3469 cases of AS). Owing to a small number of participants who withdrew consent after our initial analysis, the composition of the cases changed slightly, but the significant change was the addition of 10 489 controls. We imputed the 1 region that contained a variant demonstrating a novel, potential association with AS (*P* ≤ 1 × 10^−6^) (eMethods in the Supplement) and reassessed the association of the variant with AS, first adjusted for age, age squared, and sex, and then further adjusted for (1) dyslipidemia, hypertension, smoking (ever or never), and diabetes; (2) the *LPA* variant rs10455872; or (3) 10 principal components in separate models. In 7 replication cohorts totaling 256 926 participants (5926 cases of AS) (eTable 2 in the Supplement), we estimated the association of this variant with AS. To assess the overall association of the variant with AS, we performed a fixed-effects meta-analysis using estimates from discovery and replication cohorts, weighted by the inverse of their variance. As a sensitivity analysis, we performed a fixed-effects meta-analysis using estimates from only the replication cohorts.

In the CHARGE Consortium, we estimated the association of the variant with prevalent AVC among 6942 predominantly European participants (2245 participants with AVC). Aortic valve calcium was quantified using computed tomography and dichotomized into presence (Agatston score >0) or absence (Agatston score = 0) of AVC (eMethods in the Supplement). We also identified previously reported, genome-wide significant associations with PhenoScanner^11^ (retrieved September 23, 2018) and accessed results for this variant in a GWAS for coronary artery disease (CARDIoGRAMplusC4D consortium).^12^

### Associations of Fatty Acid Levels With AS and AVC

To investigate mediation of the lead variant through polyunsaturated fatty acid biosynthesis, we estimated the associations of 4 polyunsaturated fatty acids (arachidonic acid, linoleic acid, eicosapentaenoic acid, and α-linolenic acid) and 2 polyunsaturated fatty acid ratios reflecting fatty acid desaturation activity (ratio of arachidonic acid to linoleic acid and ratio of eicosapentaenoic acid to α-linolenic acid) with AVC in the Framingham Offspring Study (FOS) cohort (n = 1310 [492 cases of AVC]) and European-ancestry participants in the Multi-Ethnic Study of Atherosclerosis (MESA) cohort (n = 2415 [387 cases of AVC]) (eMethods in the Supplement), because measurements of fatty acid levels were unavailable in the GERA cohort. We also assessed whether the association of dietary linoleic acid or α-linolenic acid with incident AS in the Malmö Diet and Cancer Study cohort and prevalent AVC in the FOS and MESA cohorts was modified by our lead variant (eMethods in the Supplement).

### Mendelian Randomization of Arachidonic Acid, *FADS1* Expression, and *FADS2* Expression With AS and AVC

Using mendelian randomization, we estimated the association of a plasma arachidonic acid genetic risk score with AS (32 variants [eTable 3 in the Supplement]) and AVC (24 variants [eTable 4 in the Supplement]). Additional sensitivity analyses were (1) removal of the lead variant and (2) inverse variance-weighted, penalized weighted median, and Egger extension methods to assess for robustness of the findings. We also assessed whether elevated *FADS1* (OMIM 606148) and *FADS2* (OMIM 606149) expression in the liver was causally associated with prevalent AS (5 variants for *FADS1* [eTable 5 in the Supplement]) and AVC (6 variants for *FADS1* [eTable 6 in the Supplement]) using mendelian randomization. Details are provided in the eMethods in the Supplement.

### Statistical Analyses

Data were analyzed from May 1, 2017, to December 5, 2019. Genome- and locus-wide genetic associations in the GERA cohort were computed using PLINK, version 2.0.^13^ The associations of fatty acids with AVC in the MESA and FOS cohorts were estimated using R, version 3.5.1 (R Project for Statistical Computing), and SAS, version 9.4 (SAS Institute, Inc), respectively. Meta-analyses of the lead variant and mendelian randomization analyses were performed using R, version 3.5.1. Two-sided *P* ≤ 5 × 10^−8^ was deemed significant in the GWAS, and 2-sided *P* ≤ .05 was considered significant in other analyses.

## Results

### Association of the *FADS1/2* Locus With AS and AVC

**Figure 1.**
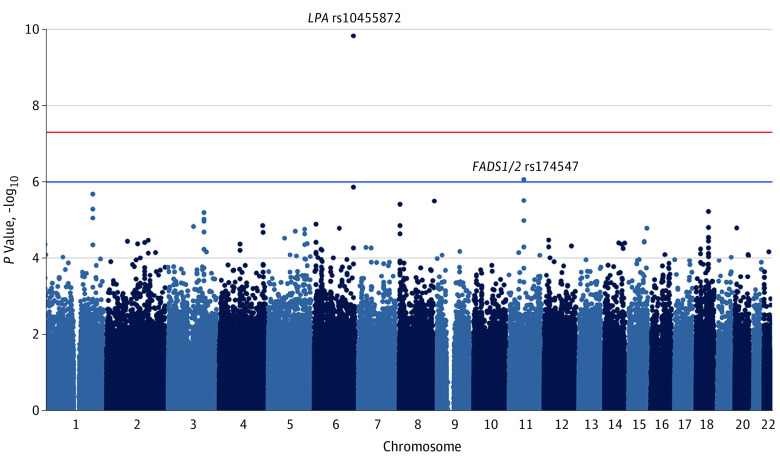
*P* Values for the Association of 615 643 Genetic Variants With Aortic Stenosis in the Genetic Epidemiology Research on Adult Health and Aging Cohort by Chromosome The red line indicates genome-wide significance (*P* ≤ 5 × 10^−8^); the blue line, suggestive evidence of association (5 × 10^−8^<*P* ≤ 1 × 10^−6^).

The discovery cohort of 44 703 participants consisted of 22 019 men (49.3%) and 22 684 women (50.7%), with a mean (SD) age of 69.7 (8.4) years. We found no evidence of inflated test statistics in the GWAS for AS (genomic inflation factor, 1.03 [eFigure 2 in the Supplement]). We confirmed the associations previously reported for *LPA* and *PALMD* variants (eTable 7 in the Supplement). The intronic variant rs174547 at the *FADS1/2* (fatty acid desaturase 1 and 2) locus on chromosome 11 was the only variant that demonstrated a novel association with AS at *P* ≤ 1 × 10^−6^ (Figure 1), with each copy of the minor (C) allele (frequency, 33%) conferring 13% lower odds of AS (odds ratio [OR] per minor allele, 0.87; 95% CI, 0.83-0.92; *P* = 8.5 × 10^−7^). After imputation of the locus in the larger set of GERA participants, the association of rs174547 with AS was essentially unchanged (OR per minor allele, 0.88; 95% CI, 0.83-0.93; *P* = 3.0 × 10^−6^), with variants in high linkage disequilibrium also associated with AS (eFigure 3 in the Supplement). Further adjustment for cardiovascular risk factors, the *LPA* variant rs10455872, or population substratification did not materially change these estimates (eTable 8 in the Supplement). Participants who were homozygous for the minor allele (6182 of 55 192 [11.2%]) had 26% lower odds of AS (OR, 074; 95% CI, 0.65-0.84; *P* = 2.8 × 10^−6^) relative to participants homozygous for the major allele, adjusted for age, age squared, and sex.

**Figure 2.**
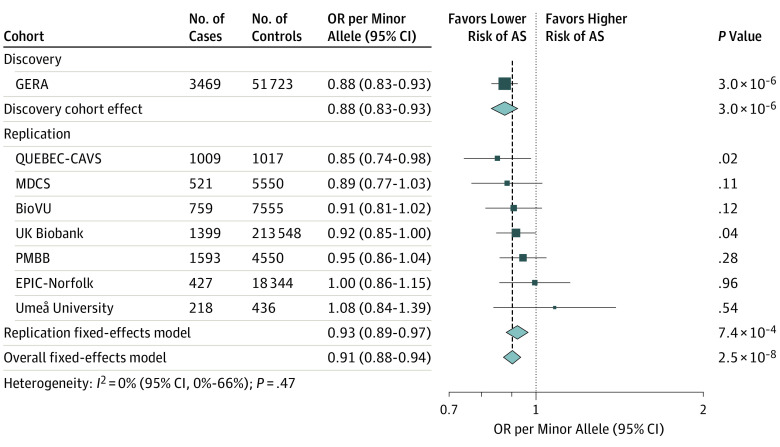
Association of *FADS1/2* rs174547 With Aortic Stenosis (AS) in the Discovery and Replication Cohorts For rs174547, C and T are the minor and major alleles, respectively. The sizes of the dark blue squares reflect the weight of the cohorts in the fixed-effects meta-analysis. BioVU indicates Vanderbilt DNA Biobank; EPIC-Norfolk, European Prospective Investigation of Cancer and Nutrition–Norfolk; GERA, Genetic Epidemiology on Adult Health and Aging; MDCS, Malmö Diet and Cancer Study; NA, not applicable; OR, odds ratio; PMBB, Penn Medicine BioBank; and QUEBEC-CAVS, Quebec City Case-Control Calcific Aortic Valve Stenosis.

When we combined this result with findings from 7 replication cohorts in a meta-analysis totaling 312 118 individuals (9395 cases of AS), the overall association reached genome-wide significance (OR per minor allele, 0.91; 95% CI, 0.88-0.94; *P* = 2.5 × 10^−8^) (Figure 2), and we observed no heterogeneity in the estimates (*I^2^* = 0%). When only the replication cohorts were meta-analyzed, the magnitude of association was similar and significant (OR per minor allele, 0.93; 95% CI, 0.89-0.97; *P* = 7.4 × 10^−4^), and there remained no heterogeneity (*I^2^* = 0%).

The rs174547 variant demonstrated a consistent association with AVC in the CHARGE consortium (OR per minor allele, 0.91; 95% CI, 0.83-0.99; *P* = .03). Results accessed from PhenoScanner (eTable 9 in the Supplement) indicated that this variant was also associated with other biochemical phenotypes, including fatty acid and lipid measures, but only 1 disease entity, asthma, which is characterized by eicosanoid-mediated inflammation. In the CARDIoGRAMplusC4D consortium, there was a nominally significant association with coronary artery disease (OR per minor allele, 0.98; 95% CI, 0.96-1.00; *P* = .03).

### Associations of Fatty Acid Levels With AS and AVC

**Figure 3.**
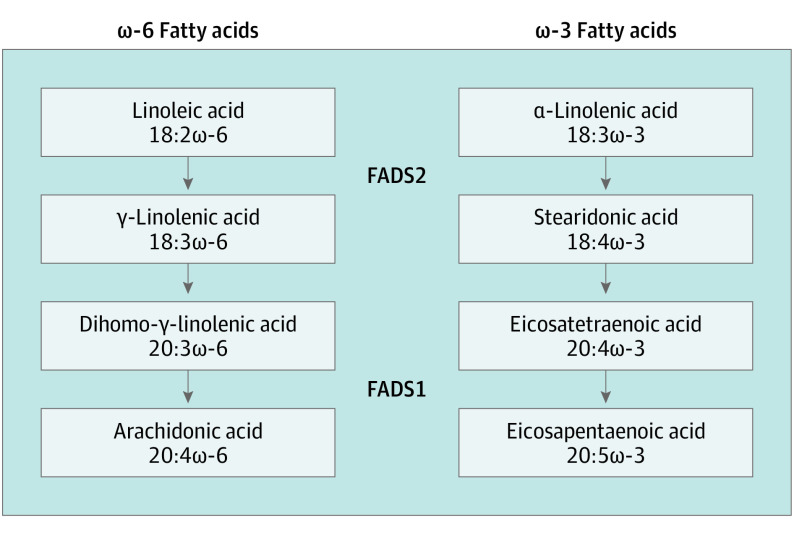
Roles of FADS1 and FADS2 in the Conversion of 18-Carbon ω-6 and ω-3 Fatty Acids to Arachidonic and Eicosapentaenoic Acids FADS1 and FADS2 perform the desaturation steps in the conversion of 18-carbon ω-6 and ω-3 fatty acids to arachidonic and eicosapentaenoic acids.

Due to the role of the *FADS1/2* locus in ω-6 and ω-3 fatty acid biosynthesis (Figure 3), we examined the association of ω-6 and ω-3 fatty acids as well as fatty acid ratios reflecting ω-6 and ω-3 desaturation activity with AVC in the FOS and MESA cohorts (Table 1 and eTable 10 in the Supplement). Higher levels of arachidonic acid and a higher ratio of arachidonic acid to linoleic acid, reflecting increased conversion of linoleic acid to arachidonic acid, were associated with increased odds for AVC, adjusted for age and sex (combined OR per SD of the natural logarithm of arachidonic acid levels, 1.12 [95% CI, 1.03-1.22; *P* = .01]; combined OR per SD of the natural logarithm of the ratio, 1.19 [95% CI, 1.09-1.30; *P* = 6.6 × 10^−5^]). These associations were materially unchanged after adjustment for low-density lipoprotein cholesterol level, systolic blood pressure, smoking, and diabetes (Table 1). Neither eicosapentaenoic acid nor the ratio of eicosapentaenoic acid to α-linolenic acid, reflecting increased conversion of α-linolenic acid to eicosapentaenoic acid, were associated with AVC. We did not observe interactions between dietary linoleic acid and rs174547 for their associations with incident AS or prevalent AVC (eTables 11 and 12 in the Supplement).

**Table 1.** Associations of ω-6 and ω-3 Fatty Acids With Aortic Valve Calcium^a^

Fatty Acid	Cohort	Adjusted for Age and Sex		Fully Adjusted^b^	
		OR (95% CI)	*P* Value	OR (95% CI)	*P* Value
ω-6					
AA level	FOS	1.10 (0.97-1.25)	.13	1.13 (0.98-1.29)	.09
	MESA	1.13 (1.01-1.27)	.04	1.14 (1.01-1.29)	.03
	Combined	1.12 (1.03-1.22)	.01	1.14 (1.04-1.24)	5.8 × 10^−3^
AA:LA ratio	FOS	1.20 (1.06-1.37)	5.2 × 10^−3^	1.22 (1.06-1.39)	4.6 × 10^−3^
	MESA	1.19 (1.06-1.34)	4.4 × 10^−3^	1.22 (1.08-1.38)	1.6 × 10^−3^
	Combined	1.19 (1.09-1.30)	6.6 × 10^−5^	1.22 (1.11-1.34)	2.2 × 10^−5^
ω-3					
EPA level	FOS	0.91 (0.80-1.04)	.16	0.91 (0.80-1.04)	.18
	MESA	1.04 (0.92-1.16)	.54	1.07 (0.95-1.21)	.25
	Combined	0.98 (0.90-1.07)	.63	1.00 (0.91-1.09)	.97
EPA:ALA ratio	FOS	0.99 (0.87-1.12)	.85	1.00 (0.88-1.15)	.95
	MESA	1.08 (0.96-1.22)	.19	1.12 (0.99-1.26)	.08
	Combined	1.04 (0.95-1.13)	.40	1.06 (0.97-1.16)	.18

Abbreviations: AA, arachidonic acid; ALA, α-linolenic acid; EPA, eicosapentaenoic acid; FOS, Framingham Offspring Study; LA, linoleic acid; MESA, Multi-Ethnic Study of Atherosclerosis; OR, odds ratio.

^a^ The ORs are calculated per SD of the natural logarithm for AA and EPA and per SD of the natural logarithm of the ratio of the fatty acids for the AA:LA and EPA:ALA ratios. Estimates were combined via fixed-effects meta-analysis weighted by the inverse of their variance.

^b^ Adjusted for low-density lipoprotein cholesterol level, systolic blood pressure, current smoking, and diabetes, in addition to age and sex.

### Mendelian Randomization of Arachidonic Acid Level and *FADS1* Expression With AS and AVC

To evaluate potential causality of ω-6 fatty acids in AS and AVC, we used mendelian randomization to estimate the associations between a plasma arachidonic acid genetic risk score and AS and AVC separately. Genetically elevated arachidonic acid level was associated with a higher prevalence of AS and AVC, with a 5–percentage point increase of arachidonic acid level among total fatty acids corresponding to an 8% increase in the odds for AS (OR, 1.08; 95% CI, 1.04-1.13; *P* = 4.1 × 10^−4^) and a 23% increase in the odds for AVC (OR, 1.23; 95% CI, 1.01-1.49; *P* = .04). In sensitivity analyses excluding the rs174547 variant or accounting for the effects of the genetic risk score through mechanisms other than arachidonic acid (ie, genetic pleiotropy), we observed attenuation under some conditions (Table 2), although the intercept in the Egger regressions was not significant.

**Table 2.** Genetic Associations of Arachidonic Acid With Aortic Stenosis and Aortic Valve Calcium

Method	OR per 5–Percentage Point Increase of AA Among Total Fatty Acids (95% CI)	*P* Value
**Aortic Stenosis**		
Mendelian randomization	1.08 (1.04-1.13)	4.1 × 10^−4^
Excluding rs174547^a^	1.03 (0.99-1.08)	.15
Inverse variance-weighted	1.08 (1.02-1.15)	8.9 × 10^−3^
Penalized weighted median	1.11 (1.03-1.20)	4.9 × 10^−3^
Egger extension to mendelian randomization	1.02 (0.92-1.13)	.72
Intercept for the Egger extension	NA	.15
**Aortic Valve Calcium**		
Mendelian randomization	1.23 (1.01-1.49)	.04
Excluding rs174547^a^	1.10 (0.82-1.49)	.52
Inverse variance-weighted	1.23 (1.01-1.49)	.04
Penalized weighted median	1.32 (1.04-1.68)	.02
Egger extension to mendelian randomization	1.08 (0.78-1.49)	.63
Intercept for the Egger extension	NA	.32

Abbreviations: AA, arachidonic acid; NA, not applicable; OR, odds ratio.

^a^ Genetic risk score is not associated with eicosapentaenoic acid.

Mendelian randomization analysis also indicated that genetically elevated *FADS1* expression in the liver conferred increased odds of AS (OR per unit increase of normalized expression, 1.31; 95% CI, 1.17-1.48; *P* = 7.4 × 10^−6^) and AVC (OR per unit increase of normalized expression, 1.25; 95% CI, 1.02-1.52; *P* = .03). Sensitivity analyses supported a potential causal association of elevated *FADS1* expression with AS and AVC, with no evidence of directional pleiotropy (eTable 13 in the Supplement). No significant *FADS2* liver expression quantitative trait loci were available.

## Discussion

We conducted a GWAS for AS in the GERA cohort, with one of the largest collections of cases of AS to date. The *FADS1/2* variant rs174547 demonstrated association with prevalent AS, with each copy of the minor allele conferring more than 10% lower odds of the disease. In individuals homozygous for the minor allele, we observed a 26% reduction in the odds of AS. The association persisted after adjustments for *LPA* rs10455872, cardiovascular risk factors, or population stratification. When the discovery and 7 replication cohorts consisting of 312 118 individuals (9395 cases of AS) were combined, rs174547 reached genome-wide significance. The association also persisted in a sensitivity analysis excluding the discovery cohort, supporting a robust association with AS. The rs174547 variant was also associated with prevalent AVC in the CHARGE consortium, providing additional evidence for a role in early valvular calcification. Because the *FADS1/2* locus is a key regulator of polyunsaturated fatty acid biosynthesis,^14^ we assessed the association of several ω-6 and ω-3 fatty acid levels with AVC. Increased production of the ω-6 arachidonic acid, but not the ω-3 eicosapentaenoic acid, was associated with AVC, with highly consistent results in 2 cohorts. We further observed that genetically elevated *FADS1* expression in the liver was associated with increased odds of AS and AVC. Additional mendelian randomization analyses provided evidence of a potentially causal association between plasma arachidonic acid, the product of the ω-6 pathway, and both AS and AVC, although we were unable to entirely exclude the possibility of pleiotropy. Therefore, our results indicate that *FADS1/*2 variation is a key determinant of valve calcification, demonstrate that plasma ω-6 fatty acids are associated with valve calcium, and suggest that increased ω-6 fatty acid biosynthesis may be a causal pathway for AS.

The rs174547 variant is located in an intron of *FADS1*, a member of the *FADS1/2/3* gene cluster. The function of *FADS3* is unknown, whereas *FADS1* and *FADS2* encode fatty acid desaturases with key functions in the conversion of dietary linoleic and α-linolenic acids into arachidonic and eicosapentaenoic acids, respectively (Figure 3).^14^ Notably, the rs174547 variant resides on a haplotype that extends across *FADS1* and part of *FADS2*, and the minor allele is associated with decreased transcription of *FADS1* and increased transcription of *FADS2* across most tissue types.^15^ The net result is lower arachidonic acid levels and higher linoleic acid levels,^16,17^ indicating that the conversion of dietary ω-6 fatty acids to longer-chain polyunsaturated fatty acids is less active in minor allele carriers. The minor allele is also associated with lower levels of eicosapentaenoic acid and higher levels of α-linolenic acid,^18^ mirroring the effects observed for ω-6 long-chain fatty acid synthesis. However, the conversion of α-linolenic acid to downstream products is inefficient, and levels of long-chain ω-3 fatty acids are highly dependent on diet.^19^ Arachidonic acid is a precursor for proinflammatory prostaglandins and leukotrienes, whereas leukotrienes and resolvins derived from eicosapentaenoic acid are anti-inflammatory,^20^ and thus the enzymatic activities of FADS1 and FADS2 proteins may have proinflammatory and anti-inflammatory effects.

Although higher linoleic acid levels have been associated with reduced risk of all-cause mortality and myocardial infarction,^21^ many prior studies have not evaluated the contribution of the ratio of arachidonic to linoleic acid (or *FADS1/2* genotypes), thereby overlooking interindividual variation in the production of arachidonic acid. A ratio of higher arachidonic acid to linoleic acid has been associated with cardiovascular and all-cause mortality after adjustment for risk factors,^22^ which suggests that FADS1 and FADS2 protein activity may independently contribute to cardiovascular outcomes.

Several lines of evidence point to ω-6 fatty acids as possible causal mediators for AS. We found that a higher ratio of arachidonic acid to linoleic acid (reflecting ω-6 desaturation), but not the ratio of eicosapentaenoic acid to α-linolenic acid (reflecting ω-3 desaturation), was associated with AVC. This finding is consistent with our mendelian randomization findings, which demonstrate that genetically elevated *FADS1* expression, as well as arachidonic acid levels, were associated with AS and AVC, providing evidence of a causal link. A greater conversion of linoleic acid to arachidonic acid may be associated with a local proinflammatory state via increased leukotrienes.^23,24^ Increased inflammation has been demonstrated among patients with AS, as denoted by overexpression of interleukin-6^25^ and interleukin-1β^26^ at the valve. Indeed, local levels of leukotrienes B_4_ and C_4_, downstream metabolites of arachidonic acid, are associated with the extent of valve calcification^27^ and aortic valve area,^25^ providing a mechanistic link between production of arachidonic acid and valve calcification and AS. Higher levels of arachidonic acid in phospholipids, observed in explanted stenotic aortic valves,^28^ may also increase their susceptibility to oxidation and promote local inflammation and subsequent calcification.

Linking variation at the *FADS1/2* locus to AS is complicated by the multitude of identified biomarker associations, which are all likely secondary to polyunsaturated fatty acid biosynthesis. The association of rs174547 with AS in the GERA cohort persisted when adjusted for dyslipidemia, hypertension, smoking, and diabetes. In the FOS and MESA cohorts, the association between AVC and greater conversion of linoleic acid to arachidonic acid also remained after adjustment for low-density lipoprotein cholesterol level, systolic blood pressure, smoking, and diabetes. Thus, the associations of rs174547 and increased arachidonic acid with aortic valve outcomes are likely to be independent of the effects of rs174547 on these risk factors. Our mendelian randomization of *FADS1* expression further demonstrates the key role of FADS1 and implicates fatty acid desaturation in valve calcification. Finally, mendelian randomization analyses for arachidonic acid were robust to pleiotropy under certain assumptions such as penalized weighted median, which allows for as many as 50% of the variants in the genetic risk score to be pleiotropic. Because the intercept did not differ significantly from zero in our Egger regression for AS or AVC, no strong evidence of alternate pleiotropic pathways was observed. Together the present findings link FADS1/2 activity with AS and identify arachidonic acid as having a likely causal association with disease. Further investigation will be needed to delineate the downstream processes that link the FADS1 and FADS2 locus and arachidonic acid to aortic valve abnormalities.

Our results point to the *FADS1/2* locus and ω-6 fatty acid biosynthesis as potential therapeutic targets. Direct therapeutic alteration of *FADS1/2* expression, to mimic the observed genetic effects and reduce fatty acid desaturation, may represent a therapeutic strategy for AS, which is supported by the results of our mendelian randomization of *FADS1* expression. Alternatively, we speculate that the role of *FADS1/2* in the conversion of linoleic acid to arachidonic acid raises the possibility of dietary modification as a preventive strategy for AS. Both approaches warrant further study as possible treatments for AS.

### Strengths and Limitations

Our discovery GWAS was performed in the GERA cohort with one of the largest collections of cases with AS assembled to date. Data from several large-scale cohorts provided robust replication for this novel association and extended it to relevant fatty acid measures. Nonetheless, we note several limitations. First, not all participants underwent echocardiography, the criterion standard for diagnosing AS. We relied on a heterogeneous definition of AS across cohorts that may not have captured all cases, but misclassification of undiagnosed AS cases as controls is likely to bias our results toward the null, as is heterogeneity in our definition of controls. Our use of diagnosis and procedure codes to define AS also precludes an assessment of disease severity. However, our case-finding approach permits the study of AS in large cohorts without echocardiographic data, has previously led to the discovery and robust replication of the *LPA* locus (including in many of the cohorts in the present study^2,4,5,9,10^), and has a positive predictive value exceeding 90%.^2^ We also observed no heterogeneity in our meta-analysis of rs174547, suggesting the various definitions for AS are concordant. Second, some mendelian randomization sensitivity analyses lacked statistical power, including Egger regression, which is a known limitation of this approach. Third, we restricted our GWAS to participants with self-reported European ancestry, because the number of non-Europeans was low and the frequency of *FADS1/2* variants varies markedly across races/ethnicities.^29^ Fourth, although all measured fatty acids were derived from blood, the measurements were taken in red blood cells in the FOS cohort and in plasma in the MESA cohort, and associations with AVC in both cohorts were cross-sectional. However, results were highly consistent across the 2 cohorts despite the different approaches. Fifth, we focused on the *FADS1/2* genes as likely candidates in the locus and provide evidence in favor of this pathway. We acknowledge that other genes nearby could also play a role, but these are unlikely candidates based on their known biology. In addition, although we observe modest reductions in the odds of AS among participants with 1 or 2 copies of the minor allele, this reflects the natural genetic variation at a locus with important biological function; targeting this locus by pharmacological means could achieve larger reductions in the odds of AS.

## Conclusions

We demonstrate that a common variant in the *FADS1/2* locus is associated with AS and AVC. Concordant findings from biomarker measurements and mendelian randomization link increased ω-6 fatty acid biosynthesis to the development of AS, which may represent a novel therapeutic target.

## Notes

Supplement. eFigure 1. Overview of the Discovery, Replication, and Follow-up Analyses Undertaken eFigure 2. Distribution of Observed Vs Expected *P* Values in the Genome-Wide Association Study for Aortic Stenosis eFigure 3. Association of Variants in the *FADS1/2* Locus With Aortic Stenosis eTable 1. Clinical Characteristics of the GERA Cohort eTable 2. Overview of Discovery and Replication Cohorts eTable 3. Variants in the Mendelian Randomization Analyses of the Association of Plasma Arachidonic Acid With Aortic Stenosis eTable 4. Variants in the Mendelian Randomization Analyses of the Association of Plasma Arachidonic Acid With Aortic Valve Calcium eTable 5. Variants in the Mendelian Randomization Analysis of the Association of Liver *FADS1* Expression With Aortic Stenosis eTable 6. Variants in the Mendelian Randomization Analysis of the Association of Liver *FADS1* Expression With Aortic Valve Calcium eTable 7. Variants Identified in Previous Genome-Wide Association Studies for Aortic Stenosis and Their Associations With Aortic Stenosis in the GERA Cohort eTable 8. Additional Covariate Adjustments for the Association of *FADS1/2* rs174547 With Aortic Stenosis in the GERA Cohort eTable 9. Associations of *FADS1/2* rs174547 With Traits at a Genome-Wide Level of Significance eTable 10. Associations of ω-6 and ω-3 Fatty Acids With Aortic Valve Calcium eTable 11. Associations of Dietary Fatty Acids With Aortic Stenosis by *FADS1/2* rs174546 Genotype in the Malmö Diet and Cancer Study eTable 12. Associations of Dietary Fatty Acids With Aortic Valve Calcium by *FADS1/2* rs174547 Genotype eTable 13. Sensitivity Analyses for the Genetic Associations of Liver *FADS1* Expression With Aortic Stenosis and Aortic Valve Calcium eFigure 1. Overview of the Discovery, Replication, and Follow-up Analyses Undertaken eFigure 2. Distribution of Observed Vs Expected *P* Values in the Genome-Wide Association Study for Aortic Stenosis eFigure 3. Association of Variants in the *FADS1/2* Locus With Aortic Stenosis eMethods. Discovery and Replication Cohorts, Aortic Valve Calcium Cohorts, Gene-Diet Interactions for Aortic Stenosis and Aortic Valve Calcium, and Genetic Associations With Aortic Stenosis and Aortic Valve Calcium eReferences.
