# Supplementary material for: Association of FADS1/2 Locus Variants and Polyunsaturated Fatty Acids With Aortic Stenosis
Source: JAMA Cardiol. 2020 Mar 18;5(6):1–9. doi: 10.1001/jamacardio.2020.0246 (PMC7081150; doi:10.1001/jamacardio.2020.0246)
Supplement: Supplement. — eFigure 1. Overview of the Discovery, Replication, and Follow-up Analyses Undertaken eFigure 2. Distribution of Observed Vs Expected P Values in the Genome-Wide Association Study for Aortic Stenosis eFigure 3. Association of Variants in the FADS1/2 Locus With Aortic Stenosis eTable 1. Clinical Characteristics of the GERA Cohort eTable 2. Overview of Discovery and Replication Cohorts eTable 3. Variants in the Mendelian Randomization Analyses of the Association of Plasma Arachidonic Acid With Aortic Stenosis eTable 4. Variants in the Mendelian Randomization Analyses of the Association of Plasma Arachidonic Acid With Aortic Valve Calcium eTable 5. Variants in the Mendelian Randomization Analysis of the Association of Liver FADS1 Expression With Aortic Stenosis eTable 6. Variants in the Mendelian Randomization Analysis of the Association of Liver FADS1 Expression With Aortic Valve Calcium eTable 7. Variants Identified in Previous Genome-Wide Association Studies for Aortic Stenosis and Their Associations With Aortic Stenosis in the GERA Cohort eTable 8. Additional Covariate Adjustments for the Association of FADS1/2 rs174547 With Aortic Stenosis in the GERA Cohort eTable 9. Associations of FADS1/2 rs174547 With Traits at a Genome-Wide Level of Significance eTable 10. Associations of ω-6 and ω-3 Fatty Acids With Aortic Valve Calcium eTable 11. Associations of Dietary Fatty Acids With Aortic Stenosis by FADS1/2 rs174546 Genotype in the Malmö Diet and Cancer Study eTable 12. Associations of Dietary Fatty Acids With Aortic Valve Calcium by FADS1/2 rs174547 Genotype eTable 13. Sensitivity Analyses for the Genetic Associations of Liver FADS1 Expression With Aortic Stenosis and Aortic Valve Calcium eFigure 1. Overview of the Discovery, Replication, and Follow-up Analyses Undertaken eFigure 2. Distribution of Observed Vs Expected P Values in the Genome-Wide Association Study for Aortic Stenosis eFigure 3. Association of Variants in the FADS1/2 Locus With Aortic Stenosis eMethods [file jamacardiol-5-694-s001.pdf]

Supplementary Online Content

Chen HY, Cairns BJ, Small AM, et al. Association of *FADS1/2* locus variants and polyunsaturated fatty acids with aortic stenosis. *JAMA Cardio*. Published online March 18, 2020. doi:10.1001/jamacardio.2020.0246

eFigure 1. Overview of the Discovery, Replication, and Follow-up Analyses Undertaken
eFigure 2. Distribution of Observed Vs Expected P Values in the Genome-Wide Association Study for Aortic Stenosis
eFigure 3. Association of Variants in the *FADS1/2* Locus With Aortic Stenosis
eTable 1. Clinical Characteristics of the GERA Cohort
eTable 2. Overview of Discovery and Replication Cohorts
eTable 3. Variants in the Mendelian Randomization Analyses of the Association of Plasma Arachidonic Acid With Aortic Stenosis
eTable 4. Variants in the Mendelian Randomization Analyses of the Association of Plasma Arachidonic Acid With Aortic Valve Calcium
eTable 5. Variants in the Mendelian Randomization Analysis of the Association of Liver *FADS1* Expression With Aortic Stenosis
eTable 6. Variants in the Mendelian Randomization Analysis of the Association of Liver *FADS1* Expression With Aortic Valve Calcium
eTable 7. Variants Identified in Previous Genome-Wide Association Studies for Aortic Stenosis and Their Associations With Aortic Stenosis in the GERA Cohort
eTable 8. Additional Covariate Adjustments for the Association of *FADS1/2* rs174547 With Aortic Stenosis in the GERA Cohort
eTable 9. Associations of *FADS1/2* rs174547 With Traits at a Genome-Wide Level of Significance
eTable 10. Associations of ω-6 and ω-3 Fatty Acids With Aortic Valve Calcium
eTable 11. Associations of Dietary Fatty Acids With Aortic Stenosis by *FADS1/2* rs174546 Genotype in the Malmö Diet and Cancer Study
eTable 12. Associations of Dietary Fatty Acids With Aortic Valve Calcium by *FADS1/2* rs174547 Genotype
eTable 13. Sensitivity Analyses for the Genetic Associations of Liver *FADS1* Expression With Aortic Stenosis and Aortic Valve Calcium
eMethods. Discovery and Replication Cohorts, Aortic Valve Calcium Cohorts, Gene-Diet Interactions for Aortic Stenosis and Aortic Valve Calcium, and Genetic Associations With Aortic Stenosis and Aortic Valve Calcium
eReferences.

This supplementary material has been provided by the authors to give readers additional information about their work.

© 2020 Chen HY et al. *JAMA Cardiol*.

eFigure 1. Overview of the Discovery, Replication, and Follow-up Analyses Undertaken

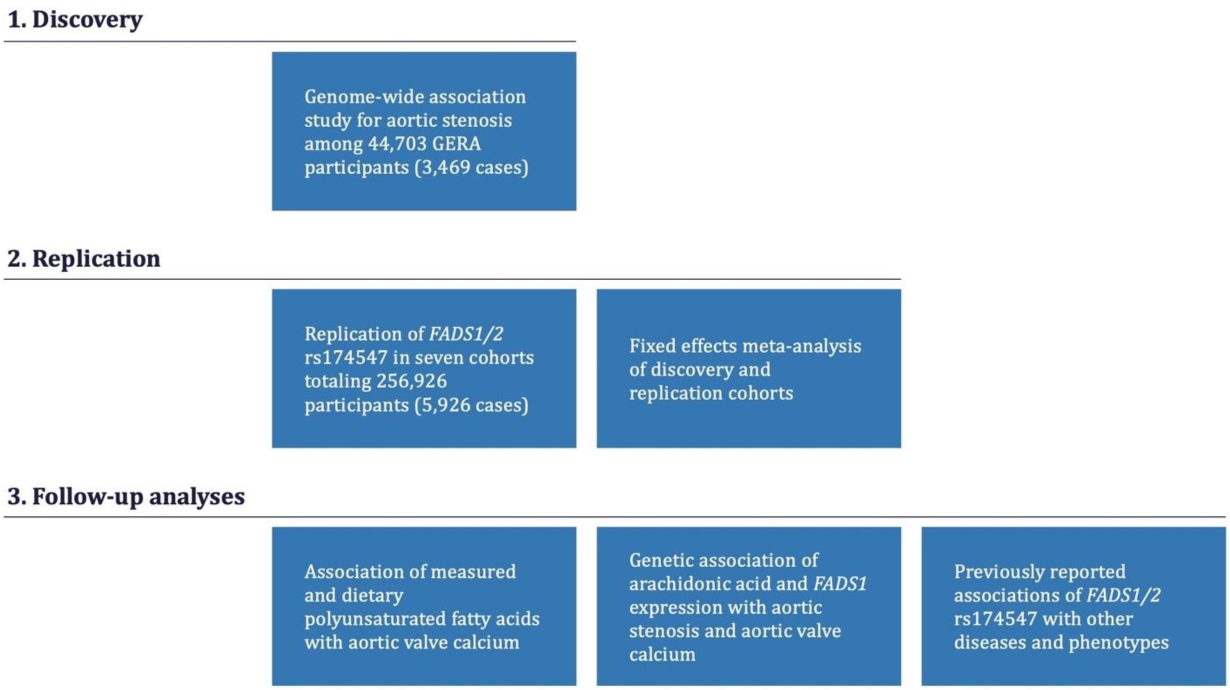

© 2020 Chen HY et al. *JAMA Cardiol*.

eFigure 2. Distribution of Observed Vs Expected *P* Values in the Genome-Wide Association Study for Aortic Stenosis

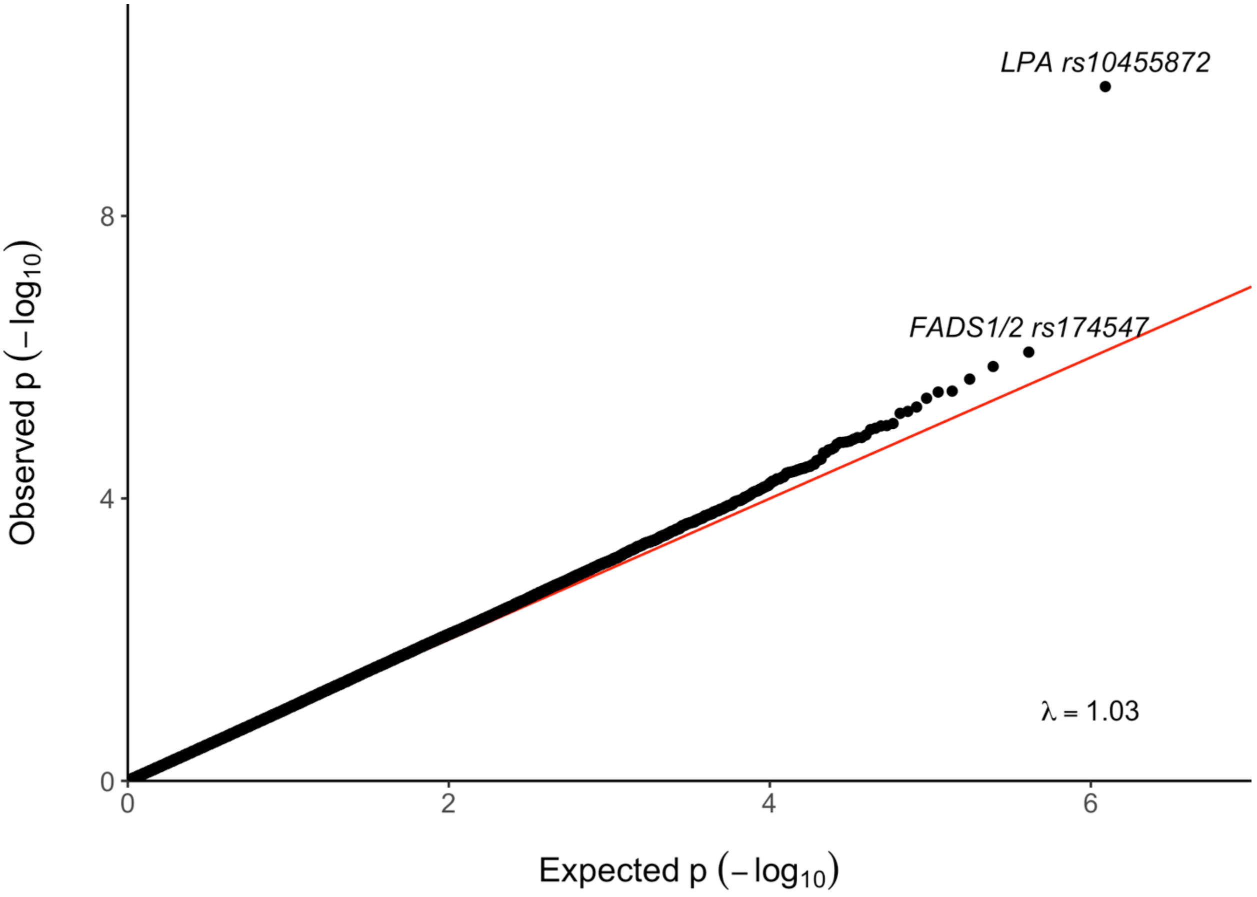

© 2020 Chen HY et al. *JAMA Cardiol*.

eFigure 3. Association of Variants in the *FADS1/2* Locus With Aortic Stenosis

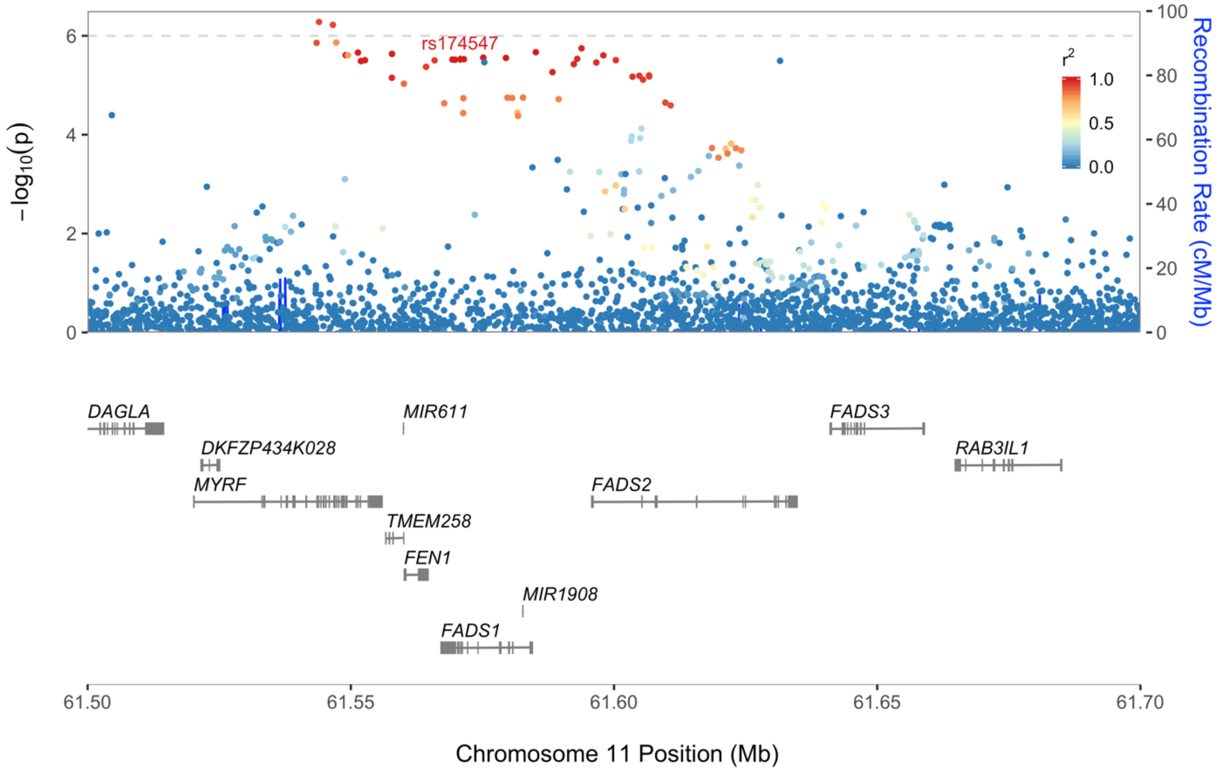

Associations with aortic stenosis were estimated following imputation of the locus among 55,192 GERA participants (3,469 cases).

Abbreviations: GERA, Genetic Epidemiology Research on Adult Health and Aging.

© 2020 Chen HY et al. *JAMA Cardiol*.

eTable 1. Clinical Characteristics of the GERA Cohort

Characteristic	Aortic Stenosis, n (%)		*p*
	Controls	Cases	
n	41,234	3,469	--
Sex, Male	20,076 (49)	1,943 (56)	<0.001
Age, Year (mean [SD])	69.3 [8.3]	74.6 [8.5]	<0.001
Body Mass Index, kg/m^2 (mean [SD])[a]	26.8 [4.9]	27.4 [5.4]	<0.001
Dyslipidemia	23,926 (58)	2,666 (77)	<0.001
Coronary Artery Disease	10,007 (24)	2,131 (61)	<0.001
Hypertension	16,961 (41)	1,945 (56)	<0.001
Ever Smoked[b]	20,247 (52)	1,834 (56)	<0.001
Diabetes	4,491 (11)	606 (17)	<0.001
Aortic Valve Replacement	0 (0)	400 (12)	--

[a] n=42,962

[b] n=42,535

Abbreviations: GERA, Genetic Epidemiology Research on Adult Health and Aging.

© 2020 Chen HY et al. *JAMA Cardiol*.

15 **eTable 2. Overview of Discovery and Replication Cohorts**

Cohort	Countries	Aortic Stenosis			Genotyping Array	Imputation Reference Panel
		No. Cases	No. Controls	Case Definition		
Discovery						
GERA[a]	United States	3,469	51,723	Electronic health records	Affymetrix Axiom	Haplotype Reference Consortium version r1.1
Replication						
QUEBEC-CAVS	Canada	1,009	1,017	Aortic valve replacement	Illumina HumanOmniExpress BeadChip	Haplotype Reference Consortium version r1.1
MDCS	Sweden	521	5,550	Electronic health records	Illumina Human Omni Express Exome BeadChip	Haplotype Reference Consortium version r1.1
BioVU	United States	759	7,555	Electronic health records	Illumina Multi-Ethnic Genotyping Array	Haplotype Reference Consortium version r1.1
UK Biobank	England, Scotland, and Wales	1,399	213,548	Electronic health records	Affymetrix Axiom	Haplotype Reference Consortium version r1.1, UK10K, and the 1000 Genomes Project phase 3
PMBB	United States	1,593	4,550	Electronic health	Illumina Quad Omni	Haplotype

© 2020 Chen HY et al. *JAMA Cardiol*.

				records	Genotyping Chip	Reference Consortium version r1.1
EPIC-Norfolk	England	427	18,344	Electronic health records and death records	Affymetrix UK Biobank Axiom Array	1000 Genomes Project phase 3
Umeå University	Sweden	218	436	Aortic valve replacement	Affymetrix UK Biobank Axiom Array r3	Haplotype Reference Consortium version r1.1

[a] Number of cases and controls after receiving updated data for the GERA cohort. The discovery genome-wide association study was conducted in an earlier dataset composed of 3,469 cases and 41,234 controls (see eTable 2).

Abbreviations: GERA, Genetic Epidemiology on Adult Health and Aging; QUEBEC-CAVS, Quebec City Case-Control Calcific Aortic Valve Stenosis; MDCS, Malmö Diet and Cancer Study; BioVU, Vanderbilt DNA Biobank; PMBB, Penn Medicine BioBank; EPIC-Norfolk, European Prospective Investigation of Cancer and Nutrition – Norfolk.

© 2020 Chen HY et al. *JAMA Cardiol*.

eTable 3. Variants in the Mendelian Randomization Analyses of the Association of Plasma Arachidonic Acid With Aortic Stenosis

Chromosome	Base Pair (GRCh37)	Genes (±50 kb)	Variant	Allele		Arachidonic Acid				Aortic Stenosis			
				Effect	Other	EAF	Beta	SE	*p*	EAF	Beta	SE	*p*
1	91,944,137	*CDC7*	rs12747494	T	C	0.99	-2.64	0.41	1.1E-10	0.95	0.04	0.04	0.28
1	102,489,339	*OLFM3*	rs11578575	T	C	0.02	2.55	0.36	1.0E-12	0.02	0.09	0.07	0.20
1	118,028,688	*MAN1A2*	rs7546429	A	G	0.98	2.75	0.15	4.0E-78	0.98	-0.07	0.07	0.29
2	99,485,920	*KIAA1211L*	rs12623171	T	C	0.01	3.20	0.45	1.9E-12	0.02	-0.07	0.06	0.21
3	41,139,829	--	rs13314643	T	C	0.01	1.44	0.15	6.3E-21	0.02	-0.02	0.07	0.75
3	119,147,958	*ARHGAP31, TMEM39A, POGLUT1*	rs16829840	T	C	0.01	0.46	0.08	2.5E-08	0.02	-0.10	0.06	0.08
3	133,893,303	*RYK*	rs11927316	A	T	0.02	0.71	0.13	4.5E-08	0.02	0.05	0.07	0.45
4	25,874,805	*SEL1L3, SMIM20*	rs11726352	A	G	0.01	2.69	0.43	3.1E-10	0.03	0.06	0.06	0.35
4	104,380,632	--	rs340480	A	G	0.98	-2.91	0.39	1.2E-13	0.96	-0.05	0.05	0.33
5	147,240,678	*SPINK1, SCGB3A2, C5orf46*	rs1432985	A	G	0.98	-2.13	0.35	1.1E-09	0.95	0.00	0.05	0.98
6	31,345,815	*HLA-B, MICA*	rs13193697	T	C	0.01	-0.72	0.13	4.1E-08	0.01	0.00	0.07	0.99
6	32,667,820	*HLA-DQB1, HLA-DQA2*	rs9275354	T	G	0.99	2.68	0.23	8.0E-31	0.77	0.07	0.02	2.7E-03
6	165,413,927	--	rs9356335	A	G	0.98	-1.16	0.10	6.1E-32	0.97	0.07	0.05	0.21

© 2020 Chen HY et al. *JAMA Cardiol.*

7	126,052,078	*GRM8*	rs7778698	A	C	0.02	-0.69	0.11	4.7E-10	0.03	-0.07	0.05	0.22
7	149,620,802	*ATP6V0E2-AS1, ATP6V0E2, DQ590227*	rs7456249	T	C	0.98	-2.68	0.41	9.4E-11	0.97	0.07	0.05	0.15
10	9,705,976	--	rs7916429	C	G	0.96	-0.51	0.09	1.3E-08	0.95	0.02	0.04	0.56
10	68,212,659	*CTNNA3, SnoU40*	rs12416578	T	C	0.99	-0.96	0.12	1.3E-14	0.99	-0.07	0.07	0.32
10	81,236,036	*ZCCHC24, AK302451, EIF5AL1*	rs7080747	T	C	0.01	-1.16	0.16	3.8E-13	0.02	-0.03	0.06	0.60
11	61,570,783	*MYRF, DKFZP434K028, BC020196, TMEM258, MIR611, FEN1, FADS1, MIR1908, FADS2*	rs174547	T	C	0.68	1.69	0.03	≈ 0	0.67	0.10	0.02	5.8E-09
11	61,823,386	--	rs11230889	A	C	0.83	0.29	0.05	9.3E-10	0.83	-0.02	0.02	0.49
11	125,259,904	*PKNOX2*	rs890455	T	G	0.02	1.85	0.32	5.1E-09	0.02	0.00	0.07	0.97
12	38,998,940	*BC040886, CPNE8*	rs2653765	A	C	0.01	-1.27	0.14	5.5E-21	0.01	-0.10	0.09	0.29
12	129,881,993	*TMEM132D*	rs7970058	A	G	0.01	-0.87	0.11	7.7E-15	0.02	-0.05	0.07	0.48
12	133,462,329	*CHFR, AK055957, ZNF605*	rs11147144	A	C	0.01	0.89	0.13	1.9E-12	0.02	0.00	0.07	0.97
13	96,870,398	*HS6ST3, Metazoa_SRP*	rs16951711	T	C	0.01	-1.80	0.13	1.6E-45	0.02	-0.08	0.06	0.18
14	20,377,949	*OR4K2, OR4K5, OR4K1*	rs4359352	A	C	0.03	2.45	0.36	7.0E-12	0.02	-0.02	0.07	0.82

© 2020 Chen HY et al. *JAMA Cardiol*.

14	20,994,759	*PNP, RNASE10, RNASE9*	rs2150349	T	C	0.02	2.01	0.35	1.1E-08	0.02	-0.04	0.07	0.58
14	41,497,960	--	rs12894905	A	C	0.02	-0.92	0.11	5.9E-17	0.02	0.01	0.05	0.80
16	15,130,351	*PDXDC1, NTAN1, RRN3*	rs1741	C	G	0.31	-0.20	0.03	1.6E-10	0.30	-0.05	0.02	2.8E-03
18	30,030,309	*GAREM*	rs17811780	T	G	0.98	-0.68	0.12	1.2E-08	0.98	0.05	0.06	0.36
18	45,488,690	*SMAD2*	rs12456907	A	C	0.03	2.37	0.29	1.5E-16	0.02	-0.02	0.07	0.82
18	68,738,240	--	rs7231821	A	T	0.99	-0.82	0.13	2.0E-10	0.98	-0.05	0.06	0.45

The effect size for arachidonic acid is expressed as the percentage point change among total plasma fatty acids while the effect size for aortic stenosis is expressed as the natural logarithm of the odds ratio. For aortic stenosis, the EAF is from the GERA cohort.

Abbreviations: EAF, effect allele frequency; GERA, Genetic Epidemiology Research on Adult Health and Aging.

© 2020 Chen HY et al. *JAMA Cardiol*.

eTable 4. Variants in the Mendelian Randomization Analyses of the Association of Plasma Arachidonic Acid With Aortic Valve Calcium

Chromosome	Base Pair (GRCh37)	Genes (±50 kb)	Variant	Allele		Arachidonic Acid				Aortic Valve Calcium			
				Effect	Other	EAF	Beta	SE	*p*	EAF	Beta	SE	*p*
1	118,028,688	*MAN1A2*	rs7546429	A	G	0.98	2.75	0.15	4.0E-78	0.96	0.01	0.15	0.93
3	41,139,829	--	rs13314643	T	C	0.01	1.44	0.15	6.3E-21	0.02	0.07	0.24	0.76
3	119,147,958	*ARHGAP31, TMEM39A, POGLUT1*	rs16829840	T	C	0.01	0.46	0.08	2.5E-08	0.02	-0.01	0.14	0.96
3	133,893,303	*RYK*	rs11927316	A	T	0.02	0.71	0.13	4.5E-08	0.02	-0.01	0.16	0.94
4	118,437,442	--	rs17862012	A	C	0.01	2.65	0.46	1.2E-08	0.01	0.51	0.33	0.13
6	31,345,815	*HLA-B, MICA*	rs13193697	T	C	0.01	-0.72	0.13	4.1E-08	0.02	-0.12	0.15	0.43
6	32,667,820	*HLA-DQB1, HLA-DQA2*	rs9275354	T	G	0.99	2.68	0.23	8.0E-31	0.99	-0.16	0.27	0.56
6	165,413,927	--	rs9356335	A	G	0.98	-1.16	0.10	6.1E-32	0.97	-0.15	0.12	0.22
7	126,052,078	*GRM8*	rs7778698	A	C	0.02	-0.69	0.11	4.7E-10	0.03	0.11	0.18	0.55
10	9,705,976	--	rs7916429	C	G	0.96	-0.51	0.09	1.3E-08	0.95	0.12	0.10	0.21
10	68,212,659	*CTNNA3, SnoU40*	rs12416578	T	C	0.99	-0.96	0.12	1.3E-14	0.98	0.13	0.17	0.44
10	81,236,036	*ZCCHC24, AK302451, EIF5AL1*	rs7080747	T	C	0.01	-1.16	0.16	3.8E-13	0.01	0.12	0.39	0.76
10	109,928,793	--	rs10082453	A	T	0.98	0.94	0.11	1.1E-18	0.98	-0.37	0.25	0.14
11	61,570,783	*MYRF, DKFZP434K028, BC020196, TMEM258, MIR611, FEN1, FADS1, MIR1908, FADS2*	rs174547	T	C	0.68	1.69	0.03	≈ 0	0.63	0.10	0.04	0.026
11	61,823,386	*NA*	rs11230889	A	C	0.83	0.29	0.05	9.3E-10	0.83	0.04	0.06	0.53

© 2020 Chen HY et al. *JAMA Cardiol*.

12	33,798,136	*NA*	rs16921193	A	T	0.01	1.06	0.11	5.4E-21	0.06	0.00	0.23	0.98
12	38,998,940	*BC040886, CPNE8*	rs2653765	A	C	0.01	-1.27	0.14	5.5E-21	0.01	0.15	0.20	0.45
12	129,881,993	*TMEM132D*	rs7970058	A	G	0.01	-0.87	0.11	7.7E-15	0.02	-0.03	0.18	0.86
12	133,462,329	*CHFR, AK055957, ZNF605*	rs11147144	A	C	0.01	0.89	0.13	1.9E-12	0.01	0.07	0.18	0.70
13	96,870,398	*HS6ST3, Metazoa_SRP*	rs16951711	T	C	0.01	-1.80	0.13	1.6E-45	0.01	-0.11	0.26	0.66
14	41,497,960	--	rs12894905	A	C	0.02	-0.92	0.11	5.9E-17	0.03	-0.07	0.13	0.58
16	15,130,351	*PDXDC1, NTAN1, RRN3*	rs1741	C	G	0.31	-0.20	0.03	1.6E-10	0.28	-0.10	0.05	0.036
18	30,030,309	*GAREM*	rs17811780	T	G	0.98	-0.68	0.12	1.2E-08	0.97	0.03	0.13	0.81
18	68,738,240	--	rs7231821	A	T	0.99	-0.82	0.13	2.0E-10	0.98	-0.06	0.25	0.80

The effect size for arachidonic acid is expressed as the percentage point change among total plasma fatty acids while the effect size for aortic valve calcium is expressed as the natural logarithm of the odds ratio.

Abbreviations: EAF, effect allele frequency.

© 2020 Chen HY et al. *JAMA Cardiol*.

eTable 5. Variants in the Mendelian Randomization Analysis of the Association of Liver *FADS1* Expression With Aortic Stenosis

Chrom.	Base Pair (GRCh37)	Variant	Allele		*FADS1* Expression			Aortic Stenosis		
			Effect	Other	Beta	SE	p	Beta	SE	P
11	61,560,081	rs174538	G	A	0.36	0.09	4.3E-05	0.13	0.03	9.3E-06
11	61,581,656	rs174559	G	A	0.39	0.09	1.9E-05	0.12	0.03	3.6E-05
11	61,581,764	rs174560	T	C	0.42	0.08	6.2E-07	0.12	0.03	4.2E-05
11	61,588,305	rs174564	A	G	0.41	0.08	4.3E-07	0.12	0.03	5.4E-06
11	61,619,829	rs174594	A	C	0.35	0.08	8.7E-06	0.10	0.03	2.9E-04

The effect size for *FADS1* gene expression is terms of the normalized effect size while the effect size for aortic stenosis is expressed as the natural logarithm of the odds ratio.

© 2020 Chen HY et al. *JAMA Cardiol*.

eTable 6. Variants in the Mendelian Randomization Analysis of the Association of Liver *FADS1* Expression With Aortic Valve Calcium

Chrom.	Base Pair (GRCh37)	Variant	Allele		*FADS1* Expression			Aortic Valve Calcium		
			**Effect**	**Other**	**Beta**	**SE**	**p**	**Beta**	**SE**	**p**
11	61,543,499	rs174528	T	C	0.37	0.08	2.5e-06	0.11	0.04	9.4e-03
11	61,560,081	rs174538	G	A	0.36	0.09	4.3e-05	0.08	0.04	0.075
11	61,564,299	rs4246215	G	T	0.35	0.08	2.7e-05	0.10	0.04	0.021
11	61,579,760	rs174555	T	C	0.41	0.09	1.8e-06	0.11	0.05	0.019
11	61,609,750	rs174583	C	T	0.39	0.08	8.7e-07	0.10	0.04	0.019
11	61,623,140	rs174601	C	T	0.34	0.08	1.7e-05	0.11	0.05	0.021

The effect size for *FADS1* gene expression is terms of the normalized effect size while the effect size for aortic valve calcium is expressed as the natural logarithm of the odds ratio.

© 2020 Chen HY et al. *JAMA Cardiol*.

eTable 7. Variants Identified in Previous Genome-Wide Association Studies for Aortic Stenosis and Their Associations With Aortic Stenosis in the GERA Cohort

**Chromosome**	**Base Pair (GRCh37)**	**Locus**	**Variant**	**Effect Allele**	**Other Allele**	**OR per Effect Allele (95% CI)**	***p***
1	100,049,785	*PALMD*	rs7543130[a]	A	C	1.06 (1.01-1.11)	0.021
2	145,825,555	*TEX41*	rs1830321[b]	T	C	1.05 (1.00-1.11)	0.054
6	161,010,118	*LPA*	rs10455872	G	A	1.34 (1.23-1.47)	1.5×10^{-10}

[a] In perfect linkage disequilibrium (r^2=1) with rs6702619 among 1000 Genomes Project European populations using LDLink[6].

[b] This estimate was for rs6725803, a variant in perfect linkage disequilibrium with rs1830321 among 1000 Genomes Project European populations using LDLink[6].

Abbreviations: GERA, Genetic Epidemiology Research on Adult Health and Aging; OR, odds ratio.

© 2020 Chen HY et al. *JAMA Cardiol*.

eTable 8. Additional Covariate Adjustments for the Association of *FADS1/2* rs174547 With Aortic Stenosis in the GERA Cohort

**Adjusted for**[a]	**No. Participants**	**OR per Minor Allele (95% CI)**	***p***
Dyslipidemia, Hypertension, Smoking, and Diabetes	52,708	0.90 (0.85-0.95)	1.1×10^{-4}
LPA rs10455872	55,192	0.88 (0.83-0.93)	3.0×10^{-6}
10 Principal Components	55,180	0.88 (0.84-0.93)	5.6×10^{-6}

[a] All models were also adjusted for age, age^2, and sex.

Abbreviations: GERA, Genetic Epidemiology Research on Adult Health and Aging.

© 2020 Chen HY et al. *JAMA Cardiol*.

eTable 9. Associations of *FADS1/2* rs174547 With Traits at a Genome-Wide Level of Significance

Trait	No. Participants	Effect Size	Standard Error	*p*	PubMed ID
**Protective Allele for Aortic Stenosis (C) Decreases Levels or Risk of Trait**					
Lipids					
Low-Density Lipoprotein	170,015	-0.0505	0.0038	7.99×10^{-38}	24097068
Total Cholesterol	184,184	-0.0472	0.0037	1.35×10^{-35}	24097068
High-Density Lipoprotein	184,044	-0.0389	0.0035	4.05×10^{-27}	24097068
Fatty Acids and Phospholipids					
Plasma Arachidonic Acid Level	-	-1.69	NA	≈ 0	24823311
Glycerophospholipid Levels	-	-0.1652	0.00585	2.00×10^{-175}	26068415
Docosapentaenoic Acid	8,866	-0.0746	0.0028	3.79×10^{-154}	21829377
Phospholipid Levels Plasma	-	-0.07	0.002647	4.00×10^{-154}	21829377
Adrenic Acid	8,631	-0.0483	0.0019	6.26×10^{-140}	24823311
Plasma Gamma-Linolenic Acid Levels	-	-0.02	0.001111	2.00×10^{-72}	24823311
Eicosapentaenoic Acid	8,866	-0.082	0.0051	1.83×10^{-57}	21829377
Stearic Acid	8,961	-0.1773	0.0193	4.42×10^{-20}	23362303
Plasma Linoleic Acid Levels	-	-0.048	0.00578	1.00×10^{-16}	26584805
Trans Fatty Acid Levels	-	-0.0032	0.0004262	6.00×10^{-14}	25646338
Sphingolipid Levels	-	-0.026	0.003765	5.00×10^{-12}	26068415
Blood Cells					
Red Cell Distribution Width	173,480	-0.04908	0.003713	6.77×10^{-40}	27863252
Mean Platelet Volume	173,480	-0.03889	0.003792	1.14×10^{-24}	27863252
Granulocyte Percentage of Myeloid White Cells	173,480	-0.03025	0.003749	7.06×10^{-16}	27863252
Granulocyte Count	173,480	-0.02593	0.003767	5.82×10^{-12}	27863252
Sum Neutrophil Eosinophil Counts	173,480	-0.02578	0.003761	7.15×10^{-12}	27863252
Eosinophil Count	173,480	-0.02407	0.003741	1.24×10^{-10}	27863252
Mean Corpuscular Volume	173,480	-0.02382	0.003704	1.26×10^{-10}	27863252

© 2020 Chen HY et al. *JAMA Cardiol*.

Sum Basophil Neutrophil Counts	173,480	-0.02375	0.003765	2.81×10^{-10}	27863252
Neutrophil Count	173,480	-0.02351	0.003758	3.94×10^{-10}	27863252
Myeloid White Cell Count	173,480	-0.02348	0.003778	5.15×10^{-10}	27863252
White Blood Cell Count	173,480	-0.0231	0.003761	8.16×10^{-10}	27863252
Sum Eosinophil Basophil Counts	173,480	-0.02255	0.003745	1.74×10^{-9}	27863252
Other					
Height	-	-0.037	0.004286	6.00×10^{-18}	25429064
Fasting Glucose	133,010	-0.019	0.0022	1.33×10^{-17}	22885924
Self-Reported Asthma	337,159	-0.005349	0.0008199	6.86×10^{-11}	UKB
**Protective Allele for Aortic Stenosis (C) Increases Levels or Risk of Trait**					
Lipids					
Triglycerides	174,696	0.0469	0.0035	1.04×10^{-40}	24097068
Fatty Acids and Phospholipids					
Linoleic Acid	8,631	1.474	0.0417	4.98×10^{-274}	24823311
Dihomo-Gamma-Linolenic Acid	8,631	0.355	0.0136	2.63×10^{-151}	24823311
Alpha-Linolenic Acid	8,866	0.0159	9.00E-04	3.47×10^{-64}	21829377
Oleic Acid	8,961	0.2282	0.0194	5.57×10^{-32}	23362303
Cis-Trans-18:2	8,013	0.0032	4.00E-04	6.17×10^{-14}	25646338
Palmitoleic Acid	8,961	0.023	0.0033	2.97×10^{-12}	23362303
Blood Cells					
Platelet Count	173,480	0.03721	0.003824	2.21×10^{-22}	27863252
Red Blood Cell Count	173,480	0.03451	0.003732	2.29×10^{-20}	27863252
Monocyte Percentage of White Cells	173,480	0.0283	0.003739	3.77×10^{-14}	27863252
Hemoglobin Concentration	173,480	0.0275	0.003721	1.47×10^{-13}	27863252
Hematocrit	173,480	0.02423	0.003705	6.16×10^{-11}	27863252
Plateletcrit	173,480	0.02208	0.003837	8.64×10^{-9}	27863252
Reticulocyte Count	173,480	0.02155	0.00377	1.09×10^{-8}	27863252
Other					
Pulse Rate	317,756	0.03383	0.002632	8.59×10^{-38}	UKB
Resting Heart Rate	-	6.2	1.034	2.00×10^{-9}	20639392

Abbreviations: UKB, UK Biobank.

© 2020 Chen HY et al. *JAMA Cardiol*.

eTable 10. Associations of ω-6 and ω-3 Fatty Acids With Aortic Valve Calcium

**Fatty Acid**	**Cohort**	**Age and Sex Adjusted**		**Fully Adjusted**	
		**OR (95% CI)**	***p***	**OR (95% CI)**	***p***
**N-6**					
LA	FOS	0.82 (0.72-0.93)	2.5×10^{-3}	0.82 (0.71-0.94)	3.5×10^{-3}
	MESA	0.85 (0.76-0.96)	8.3×10^{-3}	0.82 (0.73-0.93)	2.0×10^{-5}
	Combined	0.84 (0.77-0.91)	6.7×10^{-5}	0.82 (0.75-0.90)	2.2×10^{-5}
**N-3**					
ALA	FOS	0.91 (0.80-1.04)	0.17	0.89 (0.78-1.02)	0.093
	MESA	0.92 (0.82-1.04)	0.19	0.93 (0.82-1.05)	0.25
	Combined	0.92 (0.84-1.00)	0.058	0.91 (0.83-1.00)	0.048

OR presented per SD of the natural logarithm. Fully adjusted models were adjusted for low-density lipoprotein cholesterol, systolic blood pressure, current smoking, and diabetes, in addition to age and sex. Estimates were combined via fixed effects meta-analysis weighted by the inverse of their variance.

Abbreviations: OR, odds ratio; LA, linoleic acid; FOS, Framingham Offspring Study; MESA, Multi-Ethnic Study of Atherosclerosis; ALA, α-linolenic acid.

© 2020 Chen HY et al. *JAMA Cardiol*.

eTable 11. Associations of Dietary Fatty Acids With Aortic Stenosis by *FADS1/2* rs174546 Genotype in the Malmö Diet and Cancer Study

Fatty Acid	Hazard Ratio per SD of Natural Logarithm (95% CI)				$p_{interaction}$
	Overall	Homozygous Major	Heterozygous	Homozygous Minor	
**N-6**					
LA	1.03 (0.95-1.13)	1.14 (1.01-1.29)	0.88 (0.77-1.01)	1.08 (0.80-1.45)	0.06
**N-3**					
ALA	1.00 (0.92-1.09)	1.02 (0.90-1.16)	0.98 (0.86-1.12)	0.98 (0.73-1.33)	0.71

The rs174546 variant is in perfect linkage disequilibrium (r^2=1) with rs174547 among 1000 Genomes Project European populations using LDLink[6]. Models were adjusted for age, sex, energy intake, season, and diet method.

Abbreviations: LA, linoleic acid; ALA, α-linolenic acid.

© 2020 Chen HY et al. *JAMA Cardiol*.

eTable 12. Associations of Dietary Fatty Acids With Aortic Valve Calcium by *FADS1/2* rs174547 Genotype

Fatty Acid	Cohort	Odds Ratio per SD of Natural Logarithm (95% CI)				$p_{interaction}$
		Overall	Homozygous Major	Heterozygous	Homozygous Minor	
**N-6**						
LA	MESA	1.04 (0.92-1.18)	1.03 (0.86-1.23)	1.02 (0.83-1.25)	1.14 (0.79-1.63)	0.95
	FOS	0.86 (0.78-1.03)	1.04 (0.84-1.29)	0.80 (0.65-0.98)	0.78 (0.47-1.12)	0.065
**N-3**						
ALA	MESA	1.05 (0.93-1.19)	1.02 (0.85-1.22)	1.01 (0.83-1.23)	1.28 (0.87-1.87)	0.69
	FOS	0.86 (0.75-0.98)	0.93 (0.75-1.14)	0.79 (0.65-0.98)	0.76 (0.49-1.18)	0.29

Models were adjusted for age and sex.

Abbreviations: LA, linoleic acid; MESA, Multi-Ethnic Study of Atherosclerosis; FOS, Framingham Offspring Study; ALA, α-linolenic acid.

© 2020 Chen HY et al. *JAMA Cardiol*.

eTable 13. Sensitivity Analyses for the Genetic Associations of Liver *FADS1* Expression With Aortic Stenosis and Aortic Valve Calcium

**Method**	**Odds Ratio per Unit Increase of Normalized Expression (95% CI)**	***p***
Aortic Stenosis		
Inverse Variance-Weighted	1.31 (1.17-1.48)	7.4×10^{-6}
Penalized Weighted Median	1.35 (1.21-1.50)	4.1×10^{-8}
Egger Extension to Mendelian Randomization	1.25 (1.00-1.56)	0.054
Intercept for the Egger Extension	-	0.60
Aortic Valve Calcium		
Inverse Variance-Weighted	1.25 (1.02-1.52)	0.031
Penalized Weighted Median	1.32 (1.16-1.50)	2.2×10^{-5}
Egger Extension to Mendelian Randomization	1.02 (0.61-1.72)	0.94
Intercept for the Egger Extension	-	0.40

© 2020 Chen HY et al. *JAMA Cardiol*.

eMethods. Discovery and Replication Cohorts, Aortic Valve Calcium Cohorts, Gene-Diet Interactions for Aortic Stenosis and Aortic Valve Calcium, and Genetic Associations With Aortic Stenosis and Aortic Valve Calcium

Discovery Cohort

The Genetic Epidemiology Research on Adult Health and Aging (GERA) cohort contains more than 100,000 adults who are plan members of the Kaiser Permanente Medical Care Plan, Northern California Region and who are participants in the Kaiser Permanente Research Program on Genes, Environment, and Health. The GERA cohort features de-identified linkage of electronic health records, survey data of demographic variables, and genome-wide genotyping. The cohort is ethnically diverse and its racial composition has been previously described[1]. Genotyping of DNA extracted from saliva was performed on customized, ethnicity-specific Affymetrix Axiom arrays[2].

In the present study, we restricted our analyses to unrelated individuals of self-reported European ancestry, aged 55 years or older, free of congenital valvular heart disease (*International Classification of Diseases, Ninth Revision* [*ICD-9*] 746-747). We defined cases of aortic stenosis (AS) as individuals with either a diagnosis code for aortic stenosis (*ICD-9]* code for AS [*ICD-9* 424.1) or a procedure code for an aortic valve replacement in their electronic health records between January 1996 and December 2015, inclusive; all other participants were designated controls. This approach was validated in another health care delivery system to have a positive predictive value greater than 90%[3]. Dyslipidemic participants were identified as participants with two or more diagnoses of disorders of lipid metabolism (*ICD-9* 272) in the electronic health records and one or more statin prescriptions in the Kaiser Permanente prescriptions database. Coronary artery disease was defined as a diagnosis of myocardial

© 2020 Chen HY et al. *JAMA Cardiol*.

infarction or coronary artery disease (*ICD-9* 410-414) or a procedure code for coronary artery bypass surgery or percutaneous coronary intervention in the electronic health records, or self-reported revascularization, angina, or myocardial infarction. Hypertension, smoking, and diabetes were self-reported in questionnaire data and the body mass index was calculated from self-reported height and weight. Ages greater than 90 years had been rounded down to 90, to enhance the privacy of these participants (n=389).

Our genome-wide association study was performed among 44,703 unrelated participants (3,469 AS cases) using logistic regression models adjusted for age, age^2, and sex. We later received updated data, including genetic data for additional GERA participants and excluding participants who had withdrawn consent. We imputed the locus which contained a variant that demonstrated evidence of association with AS using the Michigan Imputation Server[4] with the Haplotype Reference Consortium version r1.1[5] as reference, among 55,192 European-ancestry GERA participants aged 55 years and older (3,469 AS cases) and our subsequent analyses were performed on this dataset.

All participants have provided written, informed consent and this study was approved by the internal review boards of Kaiser Permanente Northern California and the McGill University Health Centre Research Institute.

Replication Cohorts

All participants provided written, informed consent and each study was approved by the relevant institutional review boards.

1. Quebec City Case-Control Calcific Aortic Valve Stenosis

© 2020 Chen HY et al. *JAMA Cardiol*.

The Quebec City Case-Control Calcific Aortic Valve Stenosis cohort is an AS case-control cohort composed of 2,026 participants recruited from patients undergoing cardiac surgery at the Quebec Heart and Lung Institute. Aortic stenosis cases (n=1,009) were individuals with severe, non-rheumatic, tricuspid AS undergoing replacement of their aortic valve. Controls were patients undergoing surgery for other reasons, mostly for isolated coronary artery bypass, and were matched on age, sex, ethnicity, type 2 diabetes, and hypertension in a 1:1 scheme with the AS cases. Blood samples were genotyped using the Illumina HumanOmniExpress BeadChip and following standard quality control, 613,862 variants were used for imputation using the Michigan Imputation Server[4] with the Haplotype Reference Consortium version r.1.1[5] as the reference panel. Associations between 7,732,680 variants and AS were estimated using logistic regression models in SNPTEST version 2.5.2[7], adjusted for age, sex, and 10 principal components to account for population sub-stratification. Details of this cohort are available elsewhere[8].

2. Malmö Diet and Cancer Study

The Malmö Diet and Cancer Study is a prospective, population-based cohort of 30,447 individuals living in Malmö, Sweden. This cohort has been described previously[9], but briefly, blood samples from a nested random sub-cohort and sets of cases for a range of incident diseases collectively comprising more than half the cohort were genotyped on the Illumina Human Omni Express Exome BeadChip platform. The samples which passed genotyping quality control were imputed using the Michigan Imputation Server[4] with the Haplotype Reference Consortium version r1.1[5] as the reference panel, yielding up to 7,272,618 variants (after excluding variants with ≥5% missingness, minor allele frequency ≤0.01, and *p* for Hardy-Weinberg disequilibrium

© 2020 Chen HY et al. *JAMA Cardiol*.

<0.0001) in 16,168 individuals. AS cases were defined from nation-wide registers based on hospital diagnosis codes for AS (*International Classification of Diseases [ICD], 8th revision* 424.10, 424.11, or 424.19; *ICD-9* 424B, 424BA, or 424BB; *ICD-10* I35.0 or I35.2), with the remaining participants designated as controls. We restricted our analysis of the lead variant to 6,071 participants representing a nested randomly-selected sub-cohort (n=5,550 controls) and all incident cases of AS (n=521 AS cases), and modelled the association of the variant with AS in R version 3.5.0 using a logistic regression model adjusted for age, age^2, and sex. The association with AS of the 32 variants in the arachidonic acid (AA) genetic risk score (GRS) were estimated using PLINK 2.0 alpha[10] among 5,342 participants (n=464 AS cases) using genetic data that had been genotyped using Illumina Omni Express Exome and subsequently imputed using the Michigan Imputation Server[4] with the Haplotype Reference Consortium version r1.1[5] as the reference panel.

3. Vanderbilt DNA Biobank (BioVU)

The Vanderbilt DNA Biobank, or BioVU, is a biorepository featuring de-identified linkage of electronic health records and genetic data for patients undergoing treatment at the Vanderbilt University Medical Center. Described elsewhere[11], the biobank contains more than 225,000 DNA samples, of which 13,569 were genotyped on the Illumina Multi-Ethnic Genotyping Array (MEGA) platform. Following standard quality control, genotypes were imputed using the Haplotype Reference Consortium version r1.1 panel as reference. AS cases were defined as subjects who had at least one Transthoracic Echocardiogram (TTE) report in Vanderbilt's EHR system with an aortic stenosis severity (determined by natural language processing) of moderate, moderate-to-severe, or severe after the age of 55. Subjects with mild or

© 2020 Chen HY et al. *JAMA Cardiol*.

mild-moderate AS were not included as cases and were also excluded from the control group. Controls were defined as subjects older than 55 years with at least one TTE, but who had an aortic valve peak velocity < 1.5 m/s and no degree of AS. Among 8,314 unrelated participants of European ancestry (n=759 AS cases), we modelled the association of variants with AS in logistic regression models adjusted for age, age^2, and sex using SNPTEST version 2.5.4-beta3[7].

4. UK Biobank

The UK Biobank is a longitudinal cohort of more than 500,000 predominantly white inhabitants of the United Kingdom, aged 40-79 years at recruitment in 2006-2010. Previously described elsewhere[12], participants had provided blood samples for genotyping, completed questionnaires regarding demographic and behavioural attributes, and attended medical examinations. Genotyping of 487,409 samples had been performed on the Affymetrix Axiom platform, and following standard quality control, the genotypes were imputed using the Haplotype Reference Consortium version r1.1[5], the UK10K[13], and the 1000 Genomes Project phase 3[14] as references. Cases of AS were identified by combining EHRs for diagnosis codes for AS (*ICD-10* I35.0 or I35.2); the remaining participants were designated AS controls. We limited our analysis to 214,947 unrelated individuals of white British ancestry (n=1,399 AS cases), and using PLINK 2.0 alpha[10], assessed the association of variants with AS in logistic regression models adjusted for age, age^2, sex, recruiting centre, and 40 principal components to account for population stratification.

5. Penn Medicine BioBank

© 2020 Chen HY et al. *JAMA Cardiol*.

The Penn Medicine BioBank (PMBB) consists of multi-ethnic participants (~60,000) recruited throughout the University of Pennsylvania Health System who have consented to genotyping/sequencing and access to electronic health record phenotype data. This cohort has been described previously[15]. Cases of CAVD and disease-free controls were ascertained through electronic health record text mining, genotyping was performed using the Illumina Quad Omni Genotyping Chip, and imputation was performed using the Michigan Imputation Server[4] using the Haplotype Reference Consortium version r1.1[5] as the reference panel. AS cases were defined as the presence of a diagnosis code for AS (*ICD-9* 424.1 or *ICD-10* I35.0) or a procedure code for AVR in the EHR, or a validated procedure of text mining echocardiography reports[16]. The remaining individuals were designated AS controls. Participants with congenital heart disease (*ICD-9* 746-747 or *ICD-10* Q20-Q22) were excluded. We restricted our analysis to 6,143 European-ancestry participants (n=1,593 AS cases) and modeled the association of variants with AS in PLINK 2.0 alpha[10] using logistic regression models adjusted for age, age^2, and sex.

6. European Prospective Investigation of Cancer and Nutrition – Norfolk

The European Prospective Investigation of Cancer and Nutrition – Norfolk is a prospective, population-based cohort of 25,639 individuals living in Norfolk, United Kingdom and the construction of this cohort has been described in detail elsewhere[17]. AS cases were defined as individuals coded with AS (*ICD-10* I35) or who died with AS as an underlying cause according to their death certificate. All other participants were defined as controls. Genotyping of blood samples was performed using the Affymetrix UK Biobank Axiom Array platform and following standard quality control, imputation was performed using IMPUTE software[18] with the

© 2020 Chen HY et al. *JAMA Cardiol*.

1000 Genomes Project Phase 3[14] as the reference panel. We estimated the association of rs174547 with AS in a logistic regression model adjusted for age and sex using SPSS version 25.

7. Umeå University

The Umeå University cohort is a case-control study composed of 3,597 participants from northern Sweden who were previous participants of population-based surveys of health. Of these participants, 725 were cases who had undergone surgery for valvular heart disease and/or disease of the ascending aorta and 2,872 were controls randomly matched on a 1:4 scheme to individuals from the pool of health survey participants on the basis of age, sex, survey, questionnaire completion date, and geography[19]. Genotyping for 1,853 samples was performed on the Affymetrix UK Biobank Axiom Array r3, with 760,637 variants and 1,699 individuals passing standard genotyping quality control. We imputed using the Michigan Imputation Server[4] using the Haplotype Reference Consortium version r.1.1[5] as the reference panel. For our replication analysis, we considered only the 218 AS cases and their 436 matched controls. Since an unmatched analysis with adjustment for the matched factors can be as valid as a matched analysis[20], we modelled the association of variants with AS in unmatched, logistic regression models adjusted for age and sex in PLINK 2.0 alpha[10].

Replication of *FADS1/2* rs174547

Each cohort estimated the association of the variant with AS using logistic regression adjusted for age and sex, as well as age^2 if deemed appropriate.

© 2020 Chen HY et al. *JAMA Cardiol*.

Previously Reported Associations for *FADS1/2* rs174547

Results were obtained from PhenoScanner[21] on September 23, 2018 with a p-value cut-off of $p \leq 5 \times 10^{-8}$ (eTable 5). Associations without a direction of effect were removed, as were associations with unclear descriptions or irrelevant to disease pathology. For traits estimated in multiple studies, only the most significant association has been shown.

Aortic Valve Calcium Cohorts

Cohorts for Heart and Aging Research in Genomic Epidemiology Consortium

The Cohorts for Heart and Aging Research in Genomic Epidemiology (CHARGE) Consortium was established to identify contributors to cardiovascular and aging phenotypes through the meta-analysis of findings from large, longitudinal cohorts[22]. Among the cohorts in the CHARGE Consortium are the Multi-Ethnic Study of Atherosclerosis (MESA) and the Framingham Heart Study, which includes the Framingham Offspring Study (FOS).

1. Multi-Ethnic Study of Atherosclerosis

The MESA is a prospective study composed of 6,814 participants of white, African American, Hispanic American, and Chinese individuals, and has been previously described[23]. The cohort was established to identify the prevalence of, and contributors to, subclinical cardiovascular disease. Recruitment occurred from 2000-2002 and individuals were eligible for inclusion if they were 45-84 years, free of clinical cardiovascular disease, and living in one of six American communities (Baltimore, Maryland; Chicago, Illinois; Forsyth County, North

© 2020 Chen HY et al. *JAMA Cardiol*.

Carolina; Los Angeles County, California; Northern Manhattan and Southern Bronx, New York; and Saint Paul, Minnesota).

Fatty Acid Measurements

Lipids were extracted using a chloroform-methanol mixture from plasma, obtained at baseline, that had been stored at -70 ºC. Thin layer chromatography was used to separate cholesterol, triglycerides, and phospholipids, and fatty acid methyl esters were obtained from the phospholipid band. Separation and identification of fatty acid methyl esters were achieved using gas chromatography with a flame ionization detector. This method has been described in detail elsewhere[24].

Computed Tomography Scans

Computed tomography scans were performed using either an electron beam computed tomography (EBCT) scanner with a spatial resolution of 1.38 mm^3 (Imatron C150; General Electric Medical Systems, Milwaukee, Wisconsin, US), or a four-slice multidetector computed tomography (MDCT) scanner with a spatial resolution of 1.15 mm^3. Agatston scoring for aortic valve calcium (AVC) was performed by a single reader using proprietary offline software at a central MESA computed tomography reading centre (Los Angeles Biomedical Research Institute at Harbor-UCLA, Torrance, California, US). Details regarding the equipment used and quality control performed have been described previously[25].

Genetic Data

© 2020 Chen HY et al. *JAMA Cardiol*.

Genotyping was performed on the Affymetrix Genome-Wide Human SNP Array 6.0 (Affymetrix, Santa Clara, California, US) and following standard quality control, the genotypes were imputed using the Michigan Imputation Server[4] using the Haplotype Reference Consortium version r.1.1[5] as the reference panel.

2. Framingham Offspring Study

The FOS is a prospective study established in 1971, and consists of 5,124 individuals who were either the offspring, or the spouses of the offspring, of the original Framingham Heart Study. Details of the cohort have been described previously[26]. A majority of the FOS participants are of European descent.

Fatty Acid Measurements

Red blood cells from fasted blood, obtained during Examination 8 and previously frozen at -80 ºC, were methylated in a mixture of boron trifluoride and hexane. Fatty acid methyl esters were separated via gas chromatography and identified by comparing with a fatty acid mixture characteristic of red blood cells (GLC 727, NuCheck Prep). Details of this process have been previously reported[27].

Computed Tomography Scans

At Examination 7, two cardiac scans were performed for each participant using an eight-slice MDCT scanner (Lightspeed Ultra; General Electric Medical Systems, Milwaukee, Wisconsin, US). One of two trained readers reviewed each set of images (Aquarius workstation; TeraRecon, San Mateo, California, US), and if valve calcium was present on one or both scans,

© 2020 Chen HY et al. *JAMA Cardiol*.

the set was read by an independent, blinded, trained radiologist. AVC was then quantified in Agatston units. Interobserver agreement was high (κ=0.95)[3]. Disagreement was resolved through consensus.

Genetic Data

Genotyping was performed on the Affymetrix GeneChip Human Mapping 500K Array and 50K Human Gene Focused Panel (Affymetrix, Santa Clara, California, US). Following standard quality control, the genotypes were imputed using MaCH[28] with HapMap as the reference panel[29].

3. Associations with Aortic Valve Calcium

Prevalent Aortic Valve Calcium

A prevalent AVC variable was constructed by dichotomizing the quantitative AVC measures into no presence of AVC (Agatston score=0) or presence of AVC (Agatston score>0).

Meta-Analysis for FADS1/2 rs174547 in CHARGE Cohorts

To estimate the association of *FADS1/2* rs174547 with prevalent AVC, we performed a fixed effects meta-analysis of the FOS, MESA, and the Age, Gene/Environment Susceptibility-Reykjavik Study totalling 6,942 participants (2,245 AVC cases).

Associations Between Fatty Acids and AVC in CHARGE Cohorts

The association of four polyunsaturated fatty acids (linoleic acid, arachidonic acid, α-linolenic acid, and eicosapentaenoic acid) and two polyunsaturated fatty acid ratios (arachidonic

© 2020 Chen HY et al. *JAMA Cardiol*.

acid to linoleic acid, and eicosapentaenoic acid to α-linolenic acid) with AVC were modelled separately in MESA (2,415 European-ancestry participants [387 AVC cases]) and FOS (1,310 predominantly European participants [492 AVC cases]) using logistic regression adjusted for age and sex, with further adjustment for low-density lipoprotein cholesterol, systolic blood pressure, current smoking, and diabetes in the full models. The fatty acids were modelled per SD of the natural logarithm of the percentage of total fatty acids and the fatty acid ratios were modelled per SD of the natural logarithm of the ratio of the fatty acids. To estimate overall effects of the fatty acids on AVC, we performed fixed effects, inverse variance-weighted meta-analyses as no heterogeneity was observed across cohorts (heterogeneity $p>0.05$ for all comparisons).

Gene-Diet Interactions for Aortic Stenosis and Aortic Valve Calcium

Statistical Analysis

Among 28,041 participants with complete diet data from the MDCS, we examined whether dietary linoleic acid or α-linolenic acid intake was associated with incident AS in a Cox proportional-hazards model adjusted for age, sex, energy intake, season, and diet method. Linoleic acid intake was measured by a modified diet history methodology combining a 168-item dietary questionnaire, a 7-day menu book, and a 1-h diet history interview specifically designed for the MDCS, as previously described[30]. In 25,477 MDCS participants with available genetic data, we assessed whether this association was modified by rs174546, a variant in perfect linkage disequilibrium (r^2=1) with our lead variant, and then estimated the association of dietary LA and ALA (in percentage of energy intake) with AS in each genotypic class of the variant.

Among 2,416 MESA participants (380 AVC cases) and 1,220 FOS participants (452 AVC cases) with available food frequency data, we separately estimated the overall association

© 2020 Chen HY et al. *JAMA Cardiol*.

of dietary LA and ALA intake (in grams) with AVC. Again, both LA and ALA were natural log transformed. We also assessed whether the association between the transformed dietary LA and ALA with AVC was modified by the lead variant among participants with available genetic data (MESA: 2,416 participants, of which 380 were AVC cases; FOS: 1,149 participants, of which 427 were AVC cases), and quantified the effect of dietary LA or ALA on AVC within each genotypic class

Genetic Associations With Aortic Stenosis and Aortic Valve Calcium

Risk Score Construction

The arachidonic acid GRS was constructed from independent variants ($r^2 \leq 0.001$) associated with plasma arachidonic acid (percentage of total fatty acids) at a genome-wide level of significance ($p \leq 5 \times 10^{-8}$) in a previous publication[31]. If a variant was not available, a proxy in high linkage disequilibrium ($r^2 \geq 0.95$) was used if available.

The estimates used for aortic stenosis were obtained through meta-analysis of estimates from seven cohorts: GERA (imputed using the Michigan Imputation Server[4] with the Haplotype Reference Consortium version r1.1[5] as the reference panel), QUEBEC-CAVS, MDCS, BioVU, UKB, PMBB, and Umeå. To account for differences in minor allele frequencies, the GRS was limited to 32 variants with a minor allele frequency≥0.01 in both the AA publication and the GERA cohort. Meta-analysis was performed using PLINK version 1.9[10].

The aortic valve calcium estimates for each variant in the GRS were obtained from a prior genome-wide meta-analysis[3]. The Mendelian randomization analysis for aortic valve calcium was performed using 24 variants with available association results and a minor allele frequency≥0.01 in both the arachidonic and AVC studies.

© 2020 Chen HY et al. *JAMA Cardiol*.

Genetic Association of *FADS1* Expression with Aortic Stenosis and Aortic Valve Calcium

Risk Score Construction and Analysis

We performed Mendelian randomization to assess whether elevated *FADS1* or *FADS2* expression was causally associated with AS and AVC. Estimates for significant *FADS1* expression quantitative trait loci (eQTL) in the liver were extracted from the Gene-Tissue Expression (GTEx) Project, release V8 (dbGaP accession phs000424.v8.p2)[32]. No significant *FADS2* liver eQTL were available. Variants were included in the genetic risk score if they were non-ambiguous, bi-allelic, not in very high linkage disequilibrium with another variant in the risk score (r^2<0.9) in the GERA cohort, and an effect estimate was also available for the outcome (5 variants for *FADS1* with AS [eTable 11], 6 variants for *FADS1* with AVC [eTable 12]). The association of these variants with AS were estimated in the GERA cohort using logistic regression models adjusted for age, age^2, and sex, while their associations with AVC were extracted from a prior meta-analysis[3]. All variants were well imputed in the GERA cohort (info score≥0.3). Imputation quality metrics were not available for the AVC meta-analysis.

To estimate the causal association of *FADS1* expression with the odds of AS and AVC, we applied the inverse variance-weighted meta-analysis method of Mendelian randomization and accounted for correlation between variants in the risk score using *r* values computed in the GERA cohort using PLINK 2.0 alpha[10]. This approach to account for a correlated genetic risk score has been described previously[33]. As sensitivity analyses, we performed inverse-variance weighted, penalized weighted median, and Egger extension methods if the primary analysis was significant (eTable 13).

© 2020 Chen HY et al. *JAMA Cardiol*.

eReferences

1. Banda Y, Kvale MN, Hoffmann TJ, et al. Characterizing Race/Ethnicity and Genetic Ancestry for 100,000 Subjects in the Genetic Epidemiology Research on Adult Health and Aging (GERA) Cohort. Genetics 2015;200:1285-95.
2. Kvale MN, Hesselson S, Hoffmann TJ, et al. Genotyping Informatics and Quality Control for 100,000 Subjects in the Genetic Epidemiology Research on Adult Health and Aging (GERA) Cohort. Genetics 2015;200:1051-60.
3. Thanassoulis G, Campbell CY, Owens DS, et al. Genetic associations with valvular calcification and aortic stenosis. N Engl J Med 2013;368:503-12.
4. Das S, Forer L, Schonherr S, et al. Next-generation genotype imputation service and methods. Nat Genet 2016;48:1284-7.
5. McCarthy S, Das S, Kretzschmar W, et al. A reference panel of 64,976 haplotypes for genotype imputation. Nat Genet 2016;48:1279-83.
6. Machiela MJ, Chanock SJ. LDlink: a web-based application for exploring population-specific haplotype structure and linking correlated alleles of possible functional variants. Bioinformatics 2015;31:3555-7.
7. Marchini J, Howie B, Myers S, McVean G, Donnelly P. A new multipoint method for genome-wide association studies by imputation of genotypes. Nat Genet 2007;39:906-13.
8. Theriault S, Gaudreault N, Lamontagne M, et al. A transcriptome-wide association study identifies PALMD as a susceptibility gene for calcific aortic valve stenosis. Nat Commun 2018;9:988.
9. Smith JG, Luk K, Schulz CA, et al. Association of low-density lipoprotein cholesterol-related genetic variants with aortic valve calcium and incident aortic stenosis. JAMA 2014;312:1764-71.
10. Chang CC, Chow CC, Tellier LC, Vattikuti S, Purcell SM, Lee JJ. Second-generation PLINK: rising to the challenge of larger and richer datasets. Gigascience 2015;4:7.
11. Roden DM, Pulley JM, Basford MA, et al. Development of a large-scale de-identified DNA biobank to enable personalized medicine. Clin Pharmacol Ther 2008;84:362-9.
12. Sudlow C, Gallacher J, Allen N, et al. UK biobank: an open access resource for identifying the causes of a wide range of complex diseases of middle and old age. PLoS Med 2015;12:e1001779.
13. Huang J, Howie B, McCarthy S, et al. Improved imputation of low-frequency and rare variants using the UK10K haplotype reference panel. Nat Commun 2015;6:8111.
14. 1000 Genomes Project Consortium, Auton A, Brooks LD, et al. A global reference for human genetic variation. Nature 2015;526:68-74.
15. Dewey FE, Gusarova V, Dunbar RL, et al. Genetic and Pharmacologic Inactivation of ANGPTL3 and Cardiovascular Disease. N Engl J Med 2017;377:211-21.
16. Small AM, Kiss DH, Zlatsin Y, et al. Text mining applied to electronic cardiovascular procedure reports to identify patients with trileaflet aortic stenosis and coronary artery disease. J Biomed Inform 2017;72:77-84.
17. Day N, Oakes S, Luben R, et al. EPIC-Norfolk: study design and characteristics of the cohort. European Prospective Investigation of Cancer. Br J Cancer 1999;80 Suppl 1:95-103.
18. Howie BN, Donnelly P, Marchini J. A flexible and accurate genotype imputation method for the next generation of genome-wide association studies. PLoS Genet 2009;5:e1000529.

© 2020 Chen HY et al. *JAMA Cardiol*.

19. Ljungberg J, Johansson B, Engström KG, et al. Traditional Cardiovascular Risk Factors and Their Relation to Future Surgery for Valvular Heart Disease or Ascending Aortic Disease: A Case–Referent Study. Journal of the American Heart Association 2017;6.
20. Pearce N. Analysis of matched case-control studies. BMJ 2016;352:i969.
21. Staley JR, Blackshaw J, Kamat MA, et al. PhenoScanner: a database of human genotype-phenotype associations. Bioinformatics 2016;32:3207-9.
22. Psaty BM, O'Donnell CJ, Gudnason V, et al. Cohorts for Heart and Aging Research in Genomic Epidemiology (CHARGE) Consortium: Design of prospective meta-analyses of genome-wide association studies from 5 cohorts. Circ Cardiovasc Genet 2009;2:73-80.
23. Burke G, Lima J, Wong ND, Narula J. The Multiethnic Study of Atherosclerosis. Glob Heart 2016;11:267-8.
24. Cao J, Schwichtenberg KA, Hanson NQ, Tsai MY. Incorporation and clearance of omega-3 fatty acids in erythrocyte membranes and plasma phospholipids. Clin Chem 2006;52:2265-72.
25. Budoff MJ, Katz R, Wong ND, et al. Effect of scanner type on the reproducibility of extracoronary measures of calcification: the multi-ethnic study of atherosclerosis. Acad Radiol 2007;14:1043-9.
26. Kannel WB, Feinleib M, McNamara PM, Garrison RJ, Castelli WP. An investigation of coronary heart disease in families. The Framingham offspring study. Am J Epidemiol 1979;110:281-90.
27. Harris WS, Pottala JV, Vasan RS, Larson MG, Robins SJ. Changes in erythrocyte membrane trans and marine fatty acids between 1999 and 2006 in older Americans. J Nutr 2012;142:1297-303.
28. Li Y, Willer CJ, Ding J, Scheet P, Abecasis GR. MaCH: using sequence and genotype data to estimate haplotypes and unobserved genotypes. Genet Epidemiol 2010;34:816-34.
29. International HapMap C. A haplotype map of the human genome. Nature 2005;437:1299-320.
30. Hellstrand S, Ericson U, Gullberg B, Hedblad B, Orho-Melander M, Sonestedt E. Genetic variation in FADS1 has little effect on the association between dietary PUFA intake and cardiovascular disease. J Nutr 2014;144:1356-63.
31. Guan W, Steffen BT, Lemaitre RN, et al. Genome-wide association study of plasma N6 polyunsaturated fatty acids within the cohorts for heart and aging research in genomic epidemiology consortium. Circ Cardiovasc Genet 2014;7:321-31.
32. GTEx Consortium. Human genomics. The Genotype-Tissue Expression (GTEx) pilot analysis: multitissue gene regulation in humans. Science 2015;348:648-60.
33. Burgess S, Dudbridge F, Thompson SG. Combining information on multiple instrumental variables in Mendelian randomization: comparison of allele score and summarized data methods. Stat Med 2016;35:1880-906.

© 2020 Chen HY et al. *JAMA Cardiol*.
